# ToPS: A Framework to Manipulate Probabilistic Models of Sequence Data

**DOI:** 10.1371/journal.pcbi.1003234

**Published:** 2013-10-03

**Authors:** André Yoshiaki Kashiwabara, Ígor Bonadio, Vitor Onuchic, Felipe Amado, Rafael Mathias, Alan Mitchell Durham

**Affiliations:** 1 Graduate Program in Informatics, Federal University of Technology - Paraná, Cornélio Procópio, Paraná, Brazil; 2 Computer Science Graduate Program, Universidade de São Paulo, São Paulo, Brazil; 3 Bioinformatics Graduate Program, Universidade de São Paulo, São Paulo, Brazil; 4 Computer Science Undergraduate Program, Universidade de São Paulo, São Paulo, Brazil; 5 Department of Computer Science, Instituto de Matemática e Estatística, Universidade de São Paulo, São Paulo, Brazil; National Evolutionary Synthesis Center, United States of America

## Abstract

Discrete Markovian models can be used to characterize patterns in sequences of values and have many applications in biological sequence analysis, including gene prediction, CpG island detection, alignment, and protein profiling. We present ToPS, a computational framework that can be used to implement different applications in bioinformatics analysis by combining eight kinds of models: (i) independent and identically distributed process; (ii) variable-length Markov chain; (iii) inhomogeneous Markov chain; (iv) hidden Markov model; (v) profile hidden Markov model; (vi) pair hidden Markov model; (vii) generalized hidden Markov model; and (viii) similarity based sequence weighting. The framework includes functionality for training, simulation and decoding of the models. Additionally, it provides two methods to help parameter setting: Akaike and Bayesian information criteria (AIC and BIC). The models can be used stand-alone, combined in Bayesian classifiers, or included in more complex, multi-model, probabilistic architectures using GHMMs. In particular the framework provides a novel, flexible, implementation of decoding in GHMMs that detects when the architecture can be traversed efficiently.

This is a *PLOS Computational Biology* Software Article.

## Introduction

Markov models of nucleic acids and proteins are widely used in bioinformatics. Examples of applications include *ab initio* gene prediction [1], CpG island detection [2], protein family characterization [3], and sequence alignment [4]. Many times these models are hard coded in the analysis software, which means well-known algorithms are implemented over and over again. A system providing a wide range of these models is important to allow researchers to quickly select the most appropriate model to analyze sequences of different problem domains. In some cases, such as gene prediction, the characterization of the family of sequences may involve using various probabilistic models integrated in a single architecture.

One approach to avoid rewriting code is to use a general-purpose system such as R [5], for which there are packages for using these models [6], [7], but different packages require the use of different interfaces, which makes them harder to combine. Another alternative is a general-purpose system that can implement different models such as gHMM [8], HTK [9], HMMoC [10] and HMMConverter [11], N-SCAN [12] and Tigrscan [13] (also known as Genezilla).

HTK and gHMM have the distinctive capability of working with continuous emission distributions or, in other words, can accept sequences of arbitrary floating point numbers. HTK was designed to treat the speech recognition problem, but it can also be used to model biological sequences. However it implements only HMMs and does not provide simulations of the models. The gHMM package is a C library providing implementations for HMMs, pair-HMMs, inhomogeneous Markov chains and a mixture of PDFs. The system includes a graphical user interface and provides Python wrappers for each probabilistic model, but it does not implement GHMMs.

HMMConverter and HMMoC are systems that contain skeleton implementations of HMMs, pair-HMMs, and a generalization of the HMM where states may emit more than one symbol at a time. As a distinctive characteristic, both implement memory-efficient versions of the forward, backward, and Viterbi algorithms. However they do not implement the general GHMMs traditionally applied in gene-finding systems [13]–[16], where states emit words using a duration distribution and an arbitrary emission sub-model. In addition, they both require some familiarity with the XML language for the configuration of the models. HMMoC in particular requires also some programming language skills since the description of a model needs to include C code embedded at specific points in the XML configuration file.

Finally, N-SCAN and Tigrscan are examples of systems which implement general, configurable GHMMs that can combine different probabilistic sub-models in states with a given duration probability distribution. However, they are targeted specifically for gene prediction, offering only a restricted set of probabilistic models in a fixed architecture designed for the gene-finding problem.

In this paper we present ToPS (Toolkit for Probabilistic models of Sequences), a framework for the implementation of discrete probabilistic models for sequence data. ToPS currently implements eight kinds of models: (i) independent and identically distributed process (i.i.d); (ii) variable-length Markov chain [17]; (iii) inhomogeneous Markov chain [18]; (iv) hidden Markov model [19]; (v) profile hidden Markov model [20]; (vi) pair hidden Markov model [21]; (vii) generalized hidden Markov model (GHMM) [14]; (viii) similarity based sequence weighting (SBSW) [16]. To the best of our knowledge, ToPS is the first framework that at the same time implements this range of probabilistic models, is not restricted to any specific problem domain, and does not require from end-users any familiarity with programming languages or with the hierarchical structure of XML. Additionally, ToPS provides a novel implementation of the decoding algorithm that automatically detects GHMM architectures that can be parsed more efficiently, a characteristic that is essential for gene finders, since they have to be designed to parse long sequences. ToPS includes command-line programs for: training and simulating the models, evaluating input sequences using a specific model, performing Bayesian classification, and decoding sequences. As another novelty, ToPS includes two model selection criteria to help select the best parameters for a classification problem: Bayesian Information Criteria (BIC) [22], and Akaike Information Criteria (AIC) [23]. ToPS uses easy-to-read configuration files that describe probabilistic models in a notation close to the mathematical definitions. Finally, ToPS has an object-oriented architecture designed to facilitate extension and inclusion of new probabilistic models. Table 1 shows a comparison of the features of these general-purpose systems.

**Table 1:** Comparison of ToPS with other Markov model toolkits.

Program	Input Format	Probabilistic Models	Simulation	Distinguishing Characteristics
HMMConverter	XML	HMM	NO	memory efficient Viterbi, forward, backward
		pair-HMM		
		generalized HMM*		
HMMoC	XML	HMM	YES	memory efficient Viterbi, forward, backward
	C language	pair-HMM, triple-HMM, quad-HMM		
		generalized HMM*		
gHMM	XML	HMM	YES	continuous emission
		inhomogeneous Markov chain		graphical user interface
		pair-HMM		
		mixture of probability density functions		
HTK	XML	HMM	NO	continuous emission
Tigrscan	own language	GHMM+	NO	Does not provide Baum-Welsh training
N-SCAN	XML	GHMM+	NO	Does not provide Baum-Welsh training
**ToPS**	own language	HMM	YES	model selection criteria (AIC and BIC)
		pair-HMM		build profile-HMM from alignment
		GHMM		efficient and general GHMMs
		variable-length Markov chain		
		inhomogeneous Markov chains		
		discrete i.i.d models		
		SBSW		

* The generalized version of HMMs in HMMoC and HMMConverter is different from the GHMMs as defined by Kulp [14].

Specifically, they only allow the emission of whole words within a state, and neither allows sub models or the characterization of duration with a non-geometric distribution;

+ Tigrscan and N-SCAN implement GHMMs containing as sub-models weight arrays, maximum dependence decomposition, smoothed histograms, three-periodic Markov chains, and interpolated Markov models.

However, these models can not be used individually, as the state architecture of the GHMM is hard coded in these systems.

In this paper we describe the basic characteristics of ToPS and two examples of how to use it in practical problems: (i) a CpG island detector; (ii) a simple eukaryotic gene predictor.

The ToPS framework has been in intensive use by our research group in a wide variety of problems, including experimentation with null models [24], annotation of full transcripts, small RNA characterization and building gene predictors.

## Design and Implementation

### Architecture

ToPS was developed with an object-oriented architecture, which is important for the integration of the models in a single framework. The ToPS architecture includes three main class hierarchies: *ProbabilisticModel*, to represent model implementations; *ProbabilisticModelCreator*, to specify the on-the fly creation of models based on configuration files; and *ProbabilisticModelParameterValue*, to enable the parsing of the configuration files. These three hierarchies are used by a set of application programs that implement the framework's user functionalities (*bayes_classifier, evaluate, posterior_decoding, simulate, train, viterbi_decoding*). Implementing new models as a subclass of *ProbabilisticModel* will ensure integration with the facilities for training, simulating, decoding, integration in GHMMs and construction of Bayesian classifiers. A more detailed description of the architecture can be found in the ToPS user guide (http://tops.sourceforge.net/tops-doc.pdf).

### Model selection criteria

Many training algorithms contain parameters that can control the dimensionality of the trained model. A typical example is a Markov chain model in which the user has to choose the value of the order parameter. Another example is the Variable Length Markov Chain in which the user has to set a parameter that controls the pruning of the probabilistic suffix tree. Finding the best parameters can be a long and tedious task if it is performed by manually testing possible parameters. To aid the user with finding a good set of parameters, ToPS contains two model selection criteria that the user can specify with the training procedure:

- Bayesian Information Criteria (BIC) [22], that selects the parameters for which the corresponding model has the smallest value for the formula:
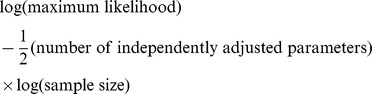

- Akaike Information Criteria (AIC) [23], that selects the parameters for which the corresponding model has the smallest value for the formula:
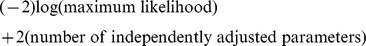
To the best of our knowledge, ToPS is currently the only framework for implementing Markovian models that provides this feature.

### Efficient decoding of GHMMs

GHMMs are very flexible probabilistic models that can be integrated with other models to describe a complex architecture. A wide majority of successful gene predictors use GHMMs as a base to recognize particular gene structures [13]–[16], [25]–[27]. However, for an unrestricted GHMM architecture that contains 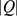 states and a sequence with length 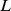, the complexity of the decoding algorithm is 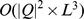 [15], [28]. This is too inefficient when we are decoding large genomic sequences in systems with many states, which is typical with gene prediction. To circumvent this problem, gene predictors impose restrictions on the GHMM's architecture in order to provide a more efficient implementation. Decoding algorithms used in gene predictors require that GHMMs satisfy three important properties (adapted from [29]):

- **Limited connectivity:** The number of transitions from a given state is less than a constant 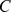. This property limits the number of previous states searched by the Viterbi algorithm, resulting in an algorithm that is in 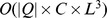.
- **Limited duration:** The states have duration distributions limited by a constant 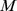. This property restricts the number of emission lengths that need to be analyzed by the Viterbi algorithm and, combined with the first restriction, results in an algorithm that is in 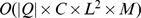.
- **Constant time lookup of the emission probabilities:** The likelihood of a subsequence can be calculated in constant time after a linear time preprocessing of the sequence, resulting, when combined with the two previous optimizations, in a decoding algorithm that is in 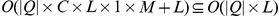.

To implement an efficient decoding algorithm, many gene-finding systems use fixed GHMM architectures hard-coded in the program and embed restrictions of the model in order to allow efficient processing. This enables efficient decoding, but limits the architectures that can be described using GHMMs and, therefore, potentially limits their applicability.

ToPS was designed for general applicability, accepting any arbitrary GHMM configurations. To do so, we introduced a methodology to automatically use efficient decoding when the architecture allows it. This is achieved by the use of an adjacency graph to represent the transitions with probability greater than zero, and by taking advantage of the object-oriented architecture of the system:

- ToPS uses a sparse graph implementation to benefit from the limited connectivity.
- The automatic detection of the constant 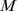 is achieved by the use of the classes representing i.i.d models which contain a list of possible durations.
- The constant time lookup of the emission probabilities is achieved by the use of the object-oriented architecture: any probabilistic model implemented as a subclass of *FactorableModel* or *InhomogeneousFactorable* represents models for which the likelihood of a sequence is factored as a product of terms, one term per sequence position. This property allows the implementation of a technique, called Prefix Sum Array [13] (PSA), that calculates the likelihood of a subsequence in constant time, after a linear time preprocessing of the sequence.

In addition, we have developed another optimization technique for the case when some observation sub-model has probability zero to emit specific words, a situation that is very common in gene-finding systems. In this case ToPS maintains an auxiliary linked list for each line of the Viterbi matrix (corresponding to the values of a given state for each position of the sequence), indicating the positions that have non-zero probability. When we have factorable models, the entries of the Viterbi matrix that generate a path with probability zero do not need to be examined. Typically, most positions have zero probability, therefore using the lists substantially reduce the running time.

These techniques achieve similar performance to the *ad-hoc* optimizations that reduce the generality of the GHMMs that can be analyzed.

## Results

**Figure 1:**
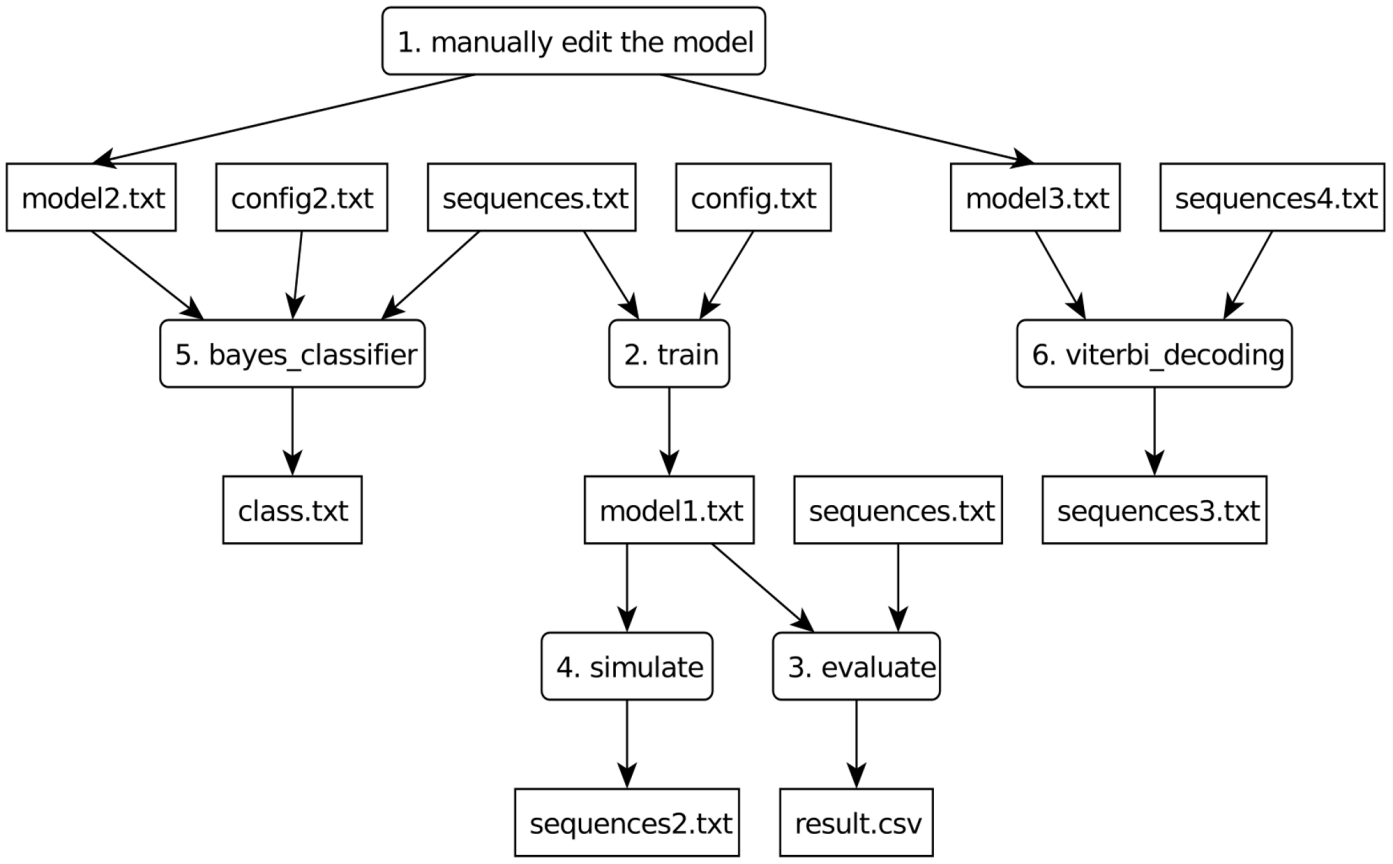
A diagram of examples of ToPS usage. Square boxes represent data files, rounded boxes represent programs or manual processes. Each model may be described manually by editing a text file (1), or the train program can be used to estimate the parameters and automatically generate such file from a training set (2). The files that contain the model parameters (in our example model1.txt, model2.txt and model3.txt) are used by the programs evaluate (3), simulate (4), bayes_classifier(5) and viterbi_decoding (6). The evaluate program calculates the likelihood of a set of input sequences given a model, the simulate program samples new sequences, the viterbi_decoding program decodes input sequences using the Viterbi algorithm, and the bayes_classifier classifies input sequences given a set of probabilistic models.

ToPS is a framework that helps describing and using discrete probabilistic models. Figure 1 illustrates the various ways to use ToPS: (i) train models given an initial specification and a set of training sequences, (ii) evaluate input sequences given a model, (iii) simulate a model, (iv) decode a sequence given a decodable model and (v) create a Bayesian classifier for sequences based on a set of pre-defined models. In this section we present two applications developed using the framework, in order to illustrate its applicability. We have chosen two well known problems in genomics: CpG island characterization and gene prediction. In both experiments we were able to improve the performance on solving the problem when comparing the ToPS implementation against published, well known alternatives.

All models, scripts, configuration files and sequence data to reproduce the experiments are available through the ToPS homepage.

### Characterizing CpG Islands with a GHMM

CpG islands (CGI) are genomic regions of great interest due to their relation with gene regulation. These regions are commonly present in the promoter region of genes. The CGI sequences typically have high G+C content with a significant high frequency of Cs followed by Gs. CGIs are also related to the DNA methylation that occurs typically at the C nucleotides. The presence of methylated DNA regions can inhibit the binding of transcription factors and therefore inhibit gene expression. Large scale experiments to detect differentially methylated regions use a CGI list as a reference, stating the importance of producing high quality CGI lists [2].

The use of Hidden Markov Model to define CGIs was described in [21] and a more accurate model in [2]. However, hidden Markov models assume that the length of each region is geometrically distributed and the observed symbols are conditionally independently distributed. With a generalized hidden Markov model we can use different models to represent CGI and non-CGI regions, and also characterize the length of CGI regions either geometrically with a self transition, or with a distribution based on known data. In this section we show how we can use these ideas in ToPS to implement CGI characterization.

**Figure 2:**
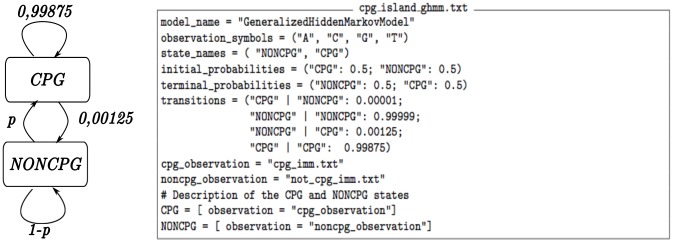
The implemented GHMM for the CpG island detector. In this GHMM we used IMMs as emission sub-models and we tested different values for the exit probability of the NONCPG state, 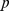, to generate the sensitivity analysis. The mean length of the CPG state emission was estimated using the training data.

Our GHMM has only two states, shown in Figure 2: CPG and NONCPG. We modeled NONCPG and CPG as states with a geometric run-length distribution represented by a self transition. To characterize both CPG and NONCPG we used Interpolated Markov Models (IMMs) [18]. IMMs have the ability of representing dependencies of arbitrary length, and we hypothesize that this model can improve CPG detection.

#### Building and training the models

To implement this system in ToPS we initially trained the two IMMs that constitute the states of the GHMM. We stored the description of these two models in the files *cpg_imm.txt* and *not_cpg_imm.txt*.

**Figure 3:**
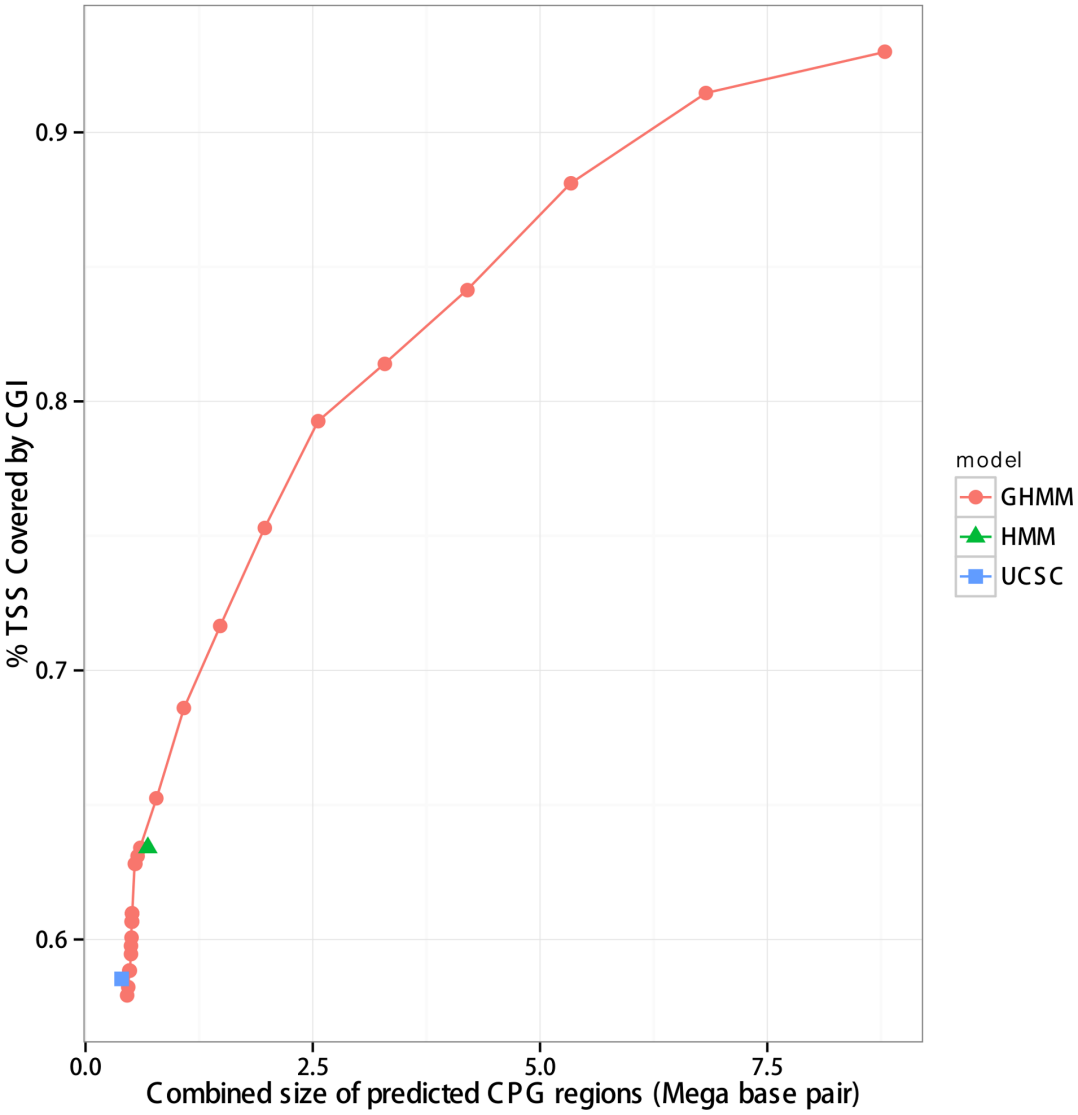
Sensitivity associated with the combined length of the predicted CGIs. In this experiment the points in the curve correspond to different values for the exit probability of the NONCPG state of the GHMM. For comparison, the results with the CGI list from UCSC Genome Browser and with the CGI list obtained using HMM [2] are shown as a blue square and green triangle, respectively.

Once we had all the trained models, we specified a GHMM with the configuration file described in the Figure 2. We assumed a mean length of 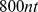 to compute the geometric duration of the CPG state. This parameter was estimated from the training data. We evaluated a set of different values of NONCPG exit probability to produce the sensitivity analysis, shown in Figure 3.

In a different experiment we evaluated another GHMM with a non-geometric duration for the CPG state (data not shown). Because the Viterbi decoding must verify the best length for the CPG state, the decoding was significantly slower than with the geometric duration GHMM (12 hours vs. 1 minute). Furthermore, we did not observe an improvement in the quality of the prediction when modeling the duration of the CPG state explicitly.

#### Evaluating the results

To characterize CpG islands and their lengths we used 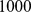 randomly chosen sequences from the CGI list of the UCSC Genome Browser [30]. The validation set was composed of 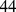 unmasked sequences corresponding to the ENCODE pilot project regions of the hg18 assembly [31].

We compared our results with two independent CGI lists: (i) the CGI list computed by an HMM developed by Wu and colleagues [2], that is stored as “Custom Annotation Tracks” in the UCSC Genome Browser; (ii) The official CGI list provided by the UCSC Genome Browser [30]. We used the comparison criteria proposed by Glass and collaborators [32], where the success of CGI prediction is measured by the rate of TSS regions covered by the CGI predictions. The TSSs were downloaded from the confirmed set of the DBTSS database [33].

As can be seen from Table 2, the GHMM results were better than those of the HMM: the ToPS GHMM (with 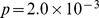) predicts 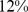 fewer nucleotides than the HMM CGI regions of Wu and collaborators (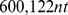 vs. 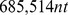), but both covered 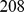 confirmed TSSs (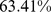 sensitivity). In a comparison against the UCSC annotation results, the GHMM (with 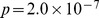) covered the same number of TSSs (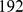), using fewer regions (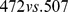). However, the GHMM predicted more nucleotides than the CGI list (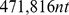 vs. 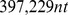), indicating that the GHMM predicted, on average, larger regions than UCSC CGI list.

**Table 2:** Comparison between CGI lists.

CGI List	Total number of CGI regions	Percentage of confirmed TSSs contained in the CGI predictions (“sensitivity”)	Total of nucleotides in CGI list (“specificity”)
UCSC Genome Browser	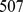	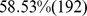	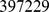
**GHMM** 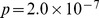	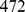	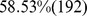	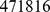
HMM [2]	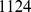	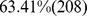	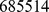
**GHMM** 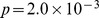	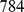	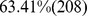	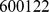

This table shows a comparison between four distinct CGI lists: the UCSC Genome Browser list, the list produced by the HMM designed by Wu and collaborators [2], and the lists produced by our GHMM approach using two distinct exit probabilities for the NONCPG state. The probabilities of the GHMM selected were those that produced lists with the same sensitivity as the ones from the UCSC Genome Browser (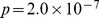), and from the HMM by Wu and collaborators (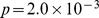).

The results obtained with different values of 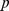 were used to generate a sensitivity analysis, as suggested by Wu and collaborators [2], shown in Figure 3. In particular, we tested the value 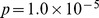 for the exit probability of the NONCPG state, which embodies the hypothesis of a CpG region for each 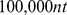, or approximately 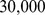 CpG regions in the human genome. This GHMM predicted 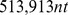 as CGI and covered 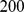 TSSs (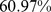 sensitivity).

### Building a protein-coding gene finder using GHMM

Predicting the location and the structure of protein-coding genes in eukaryotic genomes is a difficult but very important task [1]. To build a competitive gene finding system one is required to know a large number of non-intuitive details such as the order of each Markov model, the length of the models representing biological signals, the training set for estimating each sub-model, and the architecture of the GHMM. The majority of successful gene-finding systems uses GHMMs [13]–[16], [27], but important details are sometimes hard-coded in the the program, making it difficult to customize the GHMMs.

Next we illustrate the implementation in TopS of a gene-finding system using a GHMM with 56 states.

#### Building and training the models

**Figure 4:**
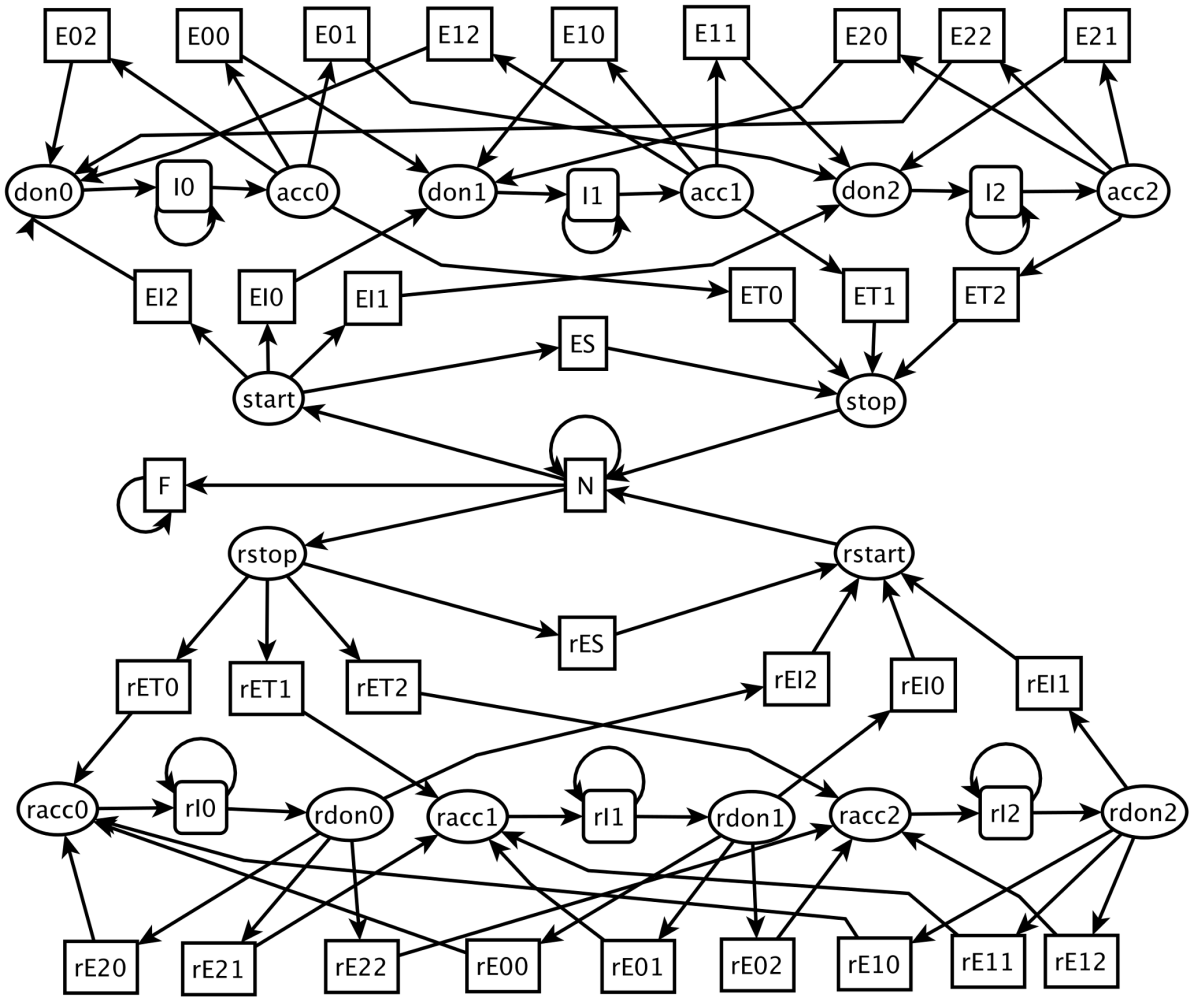
GHMM architecture for eukaryotic protein-coding gene prediction. 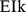
 is a state for representing an initial exon that ends at phase 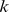. 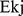 is a state for representing an internal exon that begins at phase 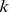 and ends at phase 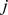. 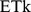 is a state for representing a terminal exon that begins at phase 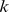. 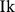 is a state for representing an intron at phase 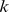. 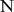 is a state for representing intergenic regions. 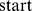 is a state for representing the start codon signal. 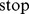 is a state for representing the stop codon signal. 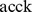 is a state for representing acceptor splice site signal at phase 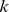. 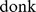 is a state for representing the donor splice site signal at phase 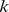. To model the reverse strand, we used the states that begin with the prefix ‘*r-*’. Squares with a self-transition represent states with geometric duration distribution. Squares without a self-transition represent states with a non-geometric duration distribution. Ellipses represent states with fixed-length durations.

The GHMM we built is shown in Figure 4. This GHMM architecture was adapted from similar GHMMs used by different gene finders and contains 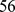 states which model genes from both DNA strands. The main differences when compared to GENSCAN [15] are the lack of states for poly-A signal and promoters in our model, and the fact that we use only one GHMM model, whereas GENSCAN uses different GHMMs for each G+C composition intervals of the target sequence.

To define a GHMM, we have to specify an emission sub-model for each state. Below is a list of the forward strand models we used:

- **start codon initial motif:** A Weight Array Model (WAM) - implemented as an inhomogeneous Markov chain - that emits a pattern of length 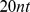 representing the sequences that appear before the start codon (ATG). We used a WAM with order estimated using BIC.
- **start codon model:** A manually edited WAM that emits the sequence “ATG” with probability 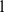.
- **initial pattern model:** A WAM that emits a pattern of length 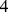 representing sequences that appear after the start codon. We used a WAM of order 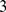.
- **stop codon model:** A WAM that emits the sequences TAA, TAG, or TGA with the same frequency distribution that appears in the training set.
- **acceptor splice site model:** A WAM that emits a pattern of length 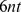 (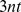 before the canonical AG, followed by the dinucleotide AG, followed by 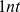 after the AG).
- **branch point model:** Windowed WAM [15] (with order 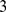 and vicinity length of 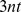) that emits a pattern of length 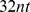 for sequences that appear before the acceptor splice site.
- **acceptor initial pattern model:** A WAM that emits a pattern of length 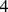 corresponding to the sequences that appear after the acceptor splice site.
- **donor splice site model:** A similarity based sequence weighting [16] representing patterns of length 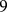 (a pattern of length 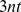, followed by the dinucleotide GT, followed by a pattern of length 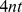).
- **donor initial pattern model:** A WAM of order 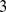 that emits a pattern of length 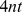 that appears before the donor splice site model emissions.
- **protein-coding model:** A three-periodic Interpolated Markov Model, with order estimated using BIC, trained with the annotated protein-coding sequences from the training set.
- **non-coding model:** An Interpolated Markov Model, with order automatically estimated using BIC, trained with the annotated 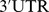 and 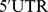 sequences.

A summarized description of each state can be found in Table 3. The states representing the reverse strand were trained with sequences corresponding to the reverse complement of those used to train the states representing the forward strand.

**Table 3:** States of the GHMM for the gene prediction problem.

State Name	Description	Emission Model	Duration Model
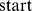	start codon	start codon initial motif (20 nt)	fixed-length (27 nt)
		start codon model (3 nt)	
		initial pattern model (4 nt)	
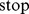	stop codon	stop codon model (3 nt)	fixed-length (3 nt)
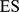	single exon	protein-coding model	Smoothed Histogram
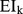	initial exons	protein-coding model	Smoothed Histogram
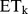	terminal exons	protein-coding model	Smoothed Histogram
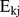	internal exon	protein-coding model	Smoothed Histogram
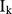	intron	non-coding model	geometric distributed
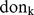	donor splice site	donor initial pattern (4 nt)	fixed-length (13 nt)
		donor splice site model (9 nt)	
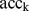	acceptor splice site	branch point model (32 nt)	fixed-length (42 nt)
		acceptor splice site model (6 nt)	
		acceptor initial pattern model (4 nt)	
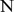	intergenic state	non-coding model	geometric distributed
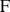	final state	non-coding model	self-transition probability is one

This table shows a summary of the configuration we used in each state of the GHMM for the gene-prediction problem. The states 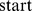, 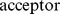, and 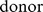 are composed of two or more individual sub-models. The reverse strand states are symmetric and were omitted from this table.

The run-length distribution of the states representing exons was trained using the same methodology described in [34] where smoothed histograms were estimated using a variation of the kernel density estimation algorithm. Because the coding segments must produce consistent gene structures, the run-length of the exon states must not allow emissions that are incompatible with their input phase and output phase. The average length of the intron sequences was estimated using the training set. Finally, the mean length of the intergenic region was estimated as 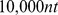.

#### Evaluating the results

To compare our results with a well established program, we applied GENSCAN [15] using the original “HumanIso.smat” parameters. As a validation set we used 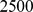 randomly selected Refseq genes from the hg18 genome obtained from the UCSC Genome Browser. We used a 5-fold cross validation experiment with our system using the “viterbi_decoding” program to decode the test sequences from each individual cross-validation run. We also applied GENSCAN to each of the five validation sets. We then used Eval [35] to calculate a set of comparative statistics, including the traditional accuracy measures for gene-finding systems. The results, shown in Table 4, indicate that ToPS achieved better performance (considering the F-score as criteria) in two measures: nucleotides (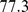 vs 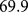) and complete gene structure (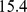 vs 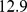). In this particular example the GHMM's performance could be probably be improved by including better models for representing short introns, and by implementing strategies to treat the C+G content variability of the genomes.

**Table 4:** Accuracy of the gene predictions.

	Gene			Exon			Nucleotide		
Predictor	PPV	S*_n_*	F-score	PPV	S*_n_*	F-score	PPV	S*_n_*	F-score
GENSCAN	9.7±1.1	19.6±0.7	12.9±1.1	54.3±2.2	**74.4±1.0**	**62.8±1.4**	55.0±4.7	**96.3±1.2**	69.9±3.7
ToPS	**12.0±1.5**	**21.6±2.2**	**15.4±1.8**	**59.0±1.8**	55.9±1.7	57.4±1.6	**69.6±5.2**	87.1±2.4	**77.3±3.1**

This table shows the accuracy of ToPS to the 5-fold cross-validation experiment. GENSCAN was tested using the “HumanIso.smat” parameters and the same test set used in each individual validation run. PPV: positive predictive value; S*_n_*: sensitivity.

### Conclusion

We presented ToPS, an open-source object-oriented framework for analyzing probabilistic models of sequence data. It implements seven well-established probabilistic models that have applications in many distinct disciplines. ToPS includes programs for simulating, decoding, classifying and evaluating discrete sequences. The implemented models can be used individually, combined in heterogeneous models using GHMMs, or integrated in Bayesian classifiers. In contrast to systems with similar goals, end users do not need any previous knowledge of programming languages, since the probabilistic models are specified using a notation close to the mathematical one. There are specific auxiliary programs for training, simulating and decoding. In addition, ToPS includes two algorithms for model selection, BIC and AIC, that can be used to find the best classification parameters for given training and validation sets. Also, in contrast to other systems, ToPS includes a GHMM implementation that is at the same time general enough to describe any GHMM architecture and efficient when the model characteristics allow for a faster version of the Viterbi algorithm. This is important to enable the use of ToPS in gene finding.

The two examples presented above, a CpG island classifier and a gene predictor, illustrate that ToPS can be used to build complex model architectures to be applied to real-world problems. In both cases we achieve competitive performance against well established results with minimal implementation work. Both results could even be improved further through experimentation with the model.

## Availability and Future Directions

ToPS was tested under GNU/Linux, and MacOSX and can be obtained from http://tops.sourceforge.net/. ToPS is distributed with a manual containing a set of examples to illustrate its use. The datasets and configuration files for the two experiments can be obtained from http://dx.doi.org/10.6084/m9.figshare.765452. Supporting information includes the source code, the manual, and a tutorial of the system (Software S1).

We are currently using ToPS to develop different probabilistic models for biological sequence analysis. In particular ToPS was useful to produce results described in [24], where we studied the problem of choosing different null-models that can reduce the number of false positives in Bayesian sequence classification. We are now developing other models for characterizing protein-coding sequences both in genomic sequences and in mRNAs, non-coding RNA characterization, and sequence aligners. In the near future, ToPS will be extended to include Maximum Dependence Decomposition models [15], Covariance Models [21] and Conditional Random Fields [36].

## Supporting Information

Software S1 Source code for ToPS. A compressed file containing the source code for ToPS.
(GZ) Click here for additional data file.
