# Supplementary material for: ToPS: A Framework to Manipulate Probabilistic Models of Sequence Data
Source: PLoS Comput Biol. 2013 Oct 3;9(10):e1003234. doi: 10.1371/journal.pcbi.1003234 (PMC3789777; doi:10.1371/journal.pcbi.1003234)
Supplement: Software S1 — Source code for ToPS. A compressed file containing the source code for ToPS. (GZ) [file pcbi.1003234.s001.gz › tops/doc/tutorial/cpg_hmm.pdf]

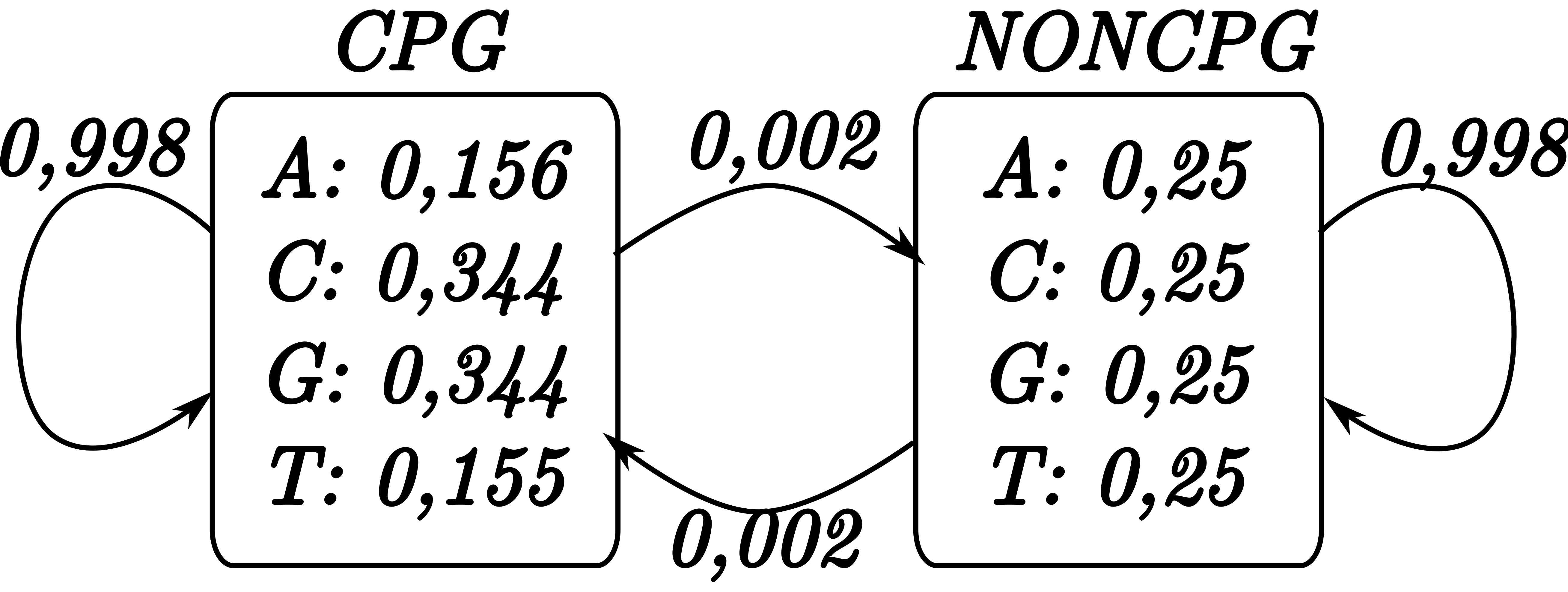
CPG
NONCPG
0,998
A: 0,156
C: 0,344
G: 0,344
T: 0,155
0,002
0,002
A: 0,25
C: 0,25
G: 0,25
T: 0,25
0,998
