# Supplementary material for: ToPS: A Framework to Manipulate Probabilistic Models of Sequence Data
Source: PLoS Comput Biol. 2013 Oct 3;9(10):e1003234. doi: 10.1371/journal.pcbi.1003234 (PMC3789777; doi:10.1371/journal.pcbi.1003234)
Supplement: Software S1 — Source code for ToPS. A compressed file containing the source code for ToPS. (GZ) [file pcbi.1003234.s001.gz › tops/doc/tutorial/tutorial.pdf]

ToPS: A framework to manipulate probabilistic models of sequence data

Kashiwabara, André Yoshiaki[1,2], Bonadio, Ígor[1], Durham, Alan Mitchell[1,*]
1 Department of Computer Science, Instituto de Matemática e Estatística, Universidade de São Paulo, São Paulo, Brazil
2 Computer engineering, Federal University of Technology, Paraná, Brazil
∗ E-mail: Corresponding aland@usp.br

Using ToPS

In this document we show how to use ToPS through examples. For the sake of brevity we present only the relevant files, the complete set is available with the download of ToPS source code. ToPS assumes that files containing input sequences are either in FASTA format (using the -F option in the command lines) or that each line starts with the sequence name, followed by a colon and the input sequence (in this section we assume the second option).

We will use the problem of modeling CpG islands in a genomic sequence with an HMM with two states [1]: one for characterizing CpG islands, another to characterize "generic" genomic sequences. We will assume that the probability of exchanging states is 0.002, and that the probability of observing the nucleotides in CpG islands is: $A : 0.156; G : 0.344; C : 0.344; T : 0.155$. Figure 1 shows the HMM.

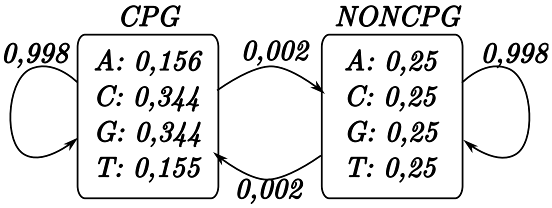

Figure 1. An illustrative example of HMM.

Manually describing probabilistic models

To specify this HMM, we need to describe a set of states, the set of observed symbols, the initial probability distribution, the transition matrix, and the probability distributions of the emissions. The cpg_island.txt file, depicted below, describes the parameters of the HMM representing the CpG island problem: (i) state_names is a list with the labels associated to states; (ii) observation_symbols is a list of arbitrary strings describing each of the observation symbols (in our case, the list of nucleotides); (iii) transitions specifies the transition probabilities between hidden states; (iv) emission_probabilities specifies the emission probabilities for each hidden state; (v) and initial_probabilities specifies the probabilities of each state being the first state in a simulation.

cpg_island.txt

```
model_name="HiddenMarkovModel"
state_names =
  ("CPG", "NONCPG" )
observation_symbols =
  ("A", "C", "G", "T")
transitions =
  ("NONCPG" | "CPG": 0.002;
```

```
   "NONCPG" | "NONCPG": 0.998
      "CPG" | "CPG": 0.998;
      "CPG" | "NONCPG": 0.002;)
emission_probabilities =
  ("A" | "CPG": 0.156;
   "C" | "CPG": 0.344;
   "G" | "CPG": 0.344;
   "T" | "CPG": 0.155;
   "A" | "NONCPG": 0.25;
   "C" | "NONCPG": 0.25;
   "G" | "NONCPG": 0.25;
   "T" | "NONCPG": 0.25)
initial_probabilities =
  ("CPG": 0.5; "NONCPG": 0.5)
```

We will use the *cpg_island.txt* file with other applications of ToPS in the next sections.

Training the models: parameter inference

If the user has annotated examples of CpG islands, then he may improve the parameters of the model by using training procedures. For HMMs, ToPS includes two training algorithms: Maximum Likelihood and Baum-Welch algorithm [2]. The Baum-Welch algorithm describes an iterative procedure that tries to find the maximum likelihood estimates of the parameters using a pre-defined HMM as the initial model, and the observed emissions as training data. With annotated sequences a good alternative is the Maximum Likelihood algorithm, which we show in our example.

To specify the details of the training procedure, we use another text file, which we will call *cpg_island_bw_train.txt*:

cpg_island_ml_train.txt

```
training_algorithm = "MaximumLikelihoodHMM"
training_set = "trainhmm_ml.sequences"
initial_specification = "cpg_island.txt"
```

The `training_set` file contains, in this case, a set of pairs of strings, the first string of each pair with the emissions and the second string with the states. The `initial_specification` file needs to include the model name, the alphabet and the states (transition and emission information is not required and, if provided, constitutes an *a priori* model).

The program *train* outputs the description of the estimated model, which the user can redirect into a file for later use. The following command line will output a specification of the model with the estimated parameters.

Command line

```
train -c cpg_island_bw_train.txt
```

If we want to use the Baum-Welch algorithm, the configuration file is similar:

cpg_island_bw_train.txt

```
training_algorithm = "BaumWelchHMM"
training_set = "trainhmm_bw.sequences"
initial_specification = "cpg_island.txt"
maxiter=300
```

The difference now is that the training set includes only strings with emission sequences (no states), and that the initial specification needs to include emission and transition probabilities. The user can also specify the maximum number of iterations of the algorithm with the *maxiter* parameter (the default is 500).

Simulating: sampling from the model

Simulation of probabilistic models is a useful procedure to illustrate and validate probabilistic models. The program *simulate*, which samples sequences from a probabilistic model, requires as parameters the length and the number of sequences to be generated. For example, the command line below determines the generation of 10 sequences, each with length 1000, using the CpG island model in standard output (screen). In this case, since we are using an HMM, the output consists of pairs of sequences (the second sequence of the pair corresponding to the hidden state labels).

Command line

```
simulate -m cpg_island.txt -n 10 -l 1000 -h
```

The command line parameters of the *simulate* program are:

- -m specifies the name of the file containing the model description.
- -n specifies the number of sequences that will be generated.
- -l specifies the length of each sequence.
- -h determines the generation of the symbols and the hidden state labels.

Decoding input sequences

With probabilistic models for which the states do not correspond to individual symbols, decoding is an essential part of the recognition problem. In ToPS, decoding uses the Viterbi algorithm [2], implemented by the program *viterbi_decoding*. In this case, the input model is an HMM or a GHMM. With the command line below, the program *viterbi_decoding* reads the file *in.txt* and, using the model specified in the file *cpg_island* generates the sequence of states visited in the most probable path of the model for each sequence. The result is presented in standard output.

Command line

```
viterbi_decoding -m cpg_island.txt < in.txt
```

The command line parameter for the *viterbi_decoding* program is:

- -m specifies the file containing the model.

Bayesian classifiers

When we have a set of pre-defined sequence families each specified by a different probabilistic model, we can use a Bayesian classifier to decide to which family a given sequence belongs. For each sequence, the Bayesian classifier selects the family that corresponds to the model with the highest posterior probability. In ToPS, the program *bayes_classifier* implements the Bayesian classifier. Based on a configuration file containing a list of specified probabilistic models and the *a priori* probabilities, this program reads sequences from the standard input and returns, for each sequence, the posterior probabilities of each

sequence name	$logP(S\|CPG)$	$P(CPG\|S)$	$logP(S\|NONCPG)$	$P(NONCPG\|S)$	classification
seq1	-141.029	0.0831703	-138.629	0.916827	NONCPG
seq2	-132.381	0.9981	-138.629	0.00192936	CPG

Table 1. An example of *bayesian_classifier*'s output.

model. In box *bayes_classifier.txt*, we show an example of such a configuration file. Using our CpG island example we can model it with two Markov chains [1], one to characterize CpG islands and another to characterize general genomic sequences. Then we can build a Bayesian classifier using the two models, and apply this classifier to candidate sequences. We will need then first to describe two Markov models, train each one, and with the trained files, build a classifier:

bayes_classifier.txt

```
classes =
 ( "CPG": "cpg_island_markov_chain.txt";
   "NONCPG": "uniform_markov_chain.txt")
model_probabilities =
 ( "CPG": 0.5;
   "NONCPG": 0.5)
```

The program reads from standard input and prints to standard output, so a sample would be:

Command line

```
bayes_classifier -c bayes_classifier.txt \
 < sequences.in
```

The program output is a table in CSV (comma separated values) format, which is compatible with most spreadsheet programs. The rows are showing, from left to right the name of each sequence, the log-likelihood of the sequence given each model, the *a posteriori* probabilities of each model, and the predicted classification of the sequence. An example of result produced by the command above is at Table 1.

GHMMs

Another situation when we need to combine probabilistic models is when we want to segment and label sequence data where different parts of a sequence are better modeled by different models. This can be accomplished with the use of GHMMs. Each state of a GHMM emits words with probabilities given by two submodels: (i) the observation model; (ii) and the duration model (the length of the observed sequence). With ToPS, the user can select any implemented probabilistic model to characterize the observation model, and use either a histogram or a geometric distribution to represent the duration distribution. Using the set of tools described in this paper, the user can train each submodel and then combine it in a GHMM architecture.

GHMMs are useful in Bioinformatics to represent the structure of genes. As an illustrative example we will use a simplified model for a bacterial gene. In bacteria, genes are regions of the genome with a different composition, specific start and stop signals, and noncoding regions separating different genes. Figure 2 illustrates this gene model. The model has four states: *NonCoding* state, representing the intergenic regions, with geometric duration distribution (represented by a self transition in the figure); *Start* and *Stop* states , representing the signals at the boundaries of a gene, with a fixed duration

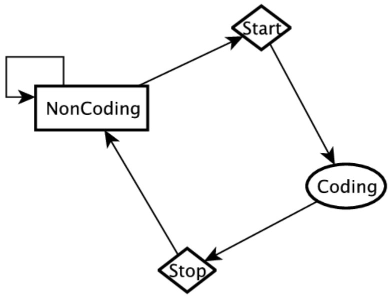

Figure 2. GHMM that represents protein-coding genes in bacteria.

distribution ; *Coding*, representing the coding region of a gene, with an i.i.d. duration distribution. Box *ghmm.txt* shows the description of the corresponding model for nonToPS.

The parameters *state_names*, *observation_symbols*, *initial_probabilities*, and *transitions* are configured in the same way as in the case of the HMM model, described above.

We have to specify the models that the GHMM will use, either by naming a file that contains its description or by inlining its description in the GHMM specification file. In our example, the GHMM uses five submodels: (i) *noncoding_model* (a *DiscreteIIDModel* inlined in the GHMM specification); (ii) *coding_model* (in file "coding.txt") ; (iii) *start_model* (in file "start.txt"); (iv) *stop_model* (in file "stop.txt"); (v) *coding_duration_model* (in file "coding_duration.txt").

After specifying the models, we have to describe the configuration of each state. ToPS assumes that the GHMM has two classes of states: (i) Fixed-length states, that emit fixed length words, and (ii) variable-length states, that emit words with lengths given by a probability distribution. There are two types of variable-length states: states with geometric distributed duration and states with non-geometric distributed duration. When specifying any state, the user have to specify the observation model using the parameter *observation*. States with geometric duration distribution are specified with a self transition, states with fixed-length dueation the user should use the parameter *sequence_length*, and other states should use the parameter *duration*.

In the file *ghmm.txt*, we have two fixed-length states (*Start*, and *Stop*) and two variable-length states (*NonCoding*, and *Coding*):

- *Start* state, with *start_model* as the observation model.
- *Stop* state, with *stop_model* as the observation model.
- *NonCoding* state, with *noncoding_model* as the observation model, and durations given by a geometric distribution in which the probability of staying in the same state is 0.999.
- *Coding* state, with *coding_model* as the observation model, and durations given by the *coding_duration_model*.

ghmm.txt

```
model_name = "GeneralizedHiddenMarkovModel"
state_names =
  ("NonCoding",
   "Start",
   "Coding",
   "Stop")
observation_symbols =
  ("A", "C", "G", "T")
```

```
initial_probabilities =
  ("NonCoding": 1.0)
transitions =
  ("NonCoding"  | "NonCoding": 0.999;
        "Start" | "NonCoding": 0.001;
       "Coding" | "Start": 1.0;
         "Stop" | "Coding": 1.0;
   "NonCoding"  | "Stop": 1.0)
noncoding_model =
  [ model_name = "DiscreteIIDModel"
    alphabet = ("A", "C", "G", "T")
    probabilities=(0.25, 0.25, 0.25, 0.25)]
coding_model = "coding.txt"
start_model = "start.txt"
stop_model = "stop.txt"
coding_duration_model="coding_duration.txt"
NonCoding =
  [ observation = noncoding_model ]
Start =
  [ observation =start_model
    sequence_length = 15 ]
Stop =
  [ observation = stop_model
    sequence_length = 15 ]
Coding =
  [ observation = coding_model
    duration = coding_duration_model ]
```

The user can use ToPS to estimate the parameters of each submodel using the *train* program. To specify the duration of non-geometric and non-fixed states, we use the *i.i.d* model with a list of sizes. After all submodels (observation , and duration) were estimated, the user can use ToPS to estimate also the transition probabilities of the GHMM. The file *train_transitions.txt* specifies the training algorithm to the *train* program:

train_transitions.txt

```
training_algorithm="GHMMTransitions"
training_set="train.txt"
ghmm_model="ghmm.txt"
```

train.txt contains the sequence of states, and *ghmm.txt* describes the initial GHMM model.

To decode a sequence with the GHMM model we use the same command we use for HMMs:

Command line

```
viterbi_decoding -m ghmm.txt < sequences.txt
```

Characterizing CpG Islands with a GHMM

CpG islands (CGI) are genomic regions of great interest due to their relation with gene regulation. These regions are commonly present in the promoter region of the genes. The CGI sequences typically have high G+C content with a significant high frequency of Cs followed by Gs. CGIs are also related to the DNA methylation that occurs typically after the C nucleotide. The presence of methylated regions can inhibit

the binding of transcription factors and therefore inhibit gene expression. Large scale experiments to detect differentially methylated regions use a CGI list as a reference, stating the importance of producing high quality CGI lists [3].

The use of Hidden Markov Model to define CGI was described in [1] and a more accurate model in [3]. However, hidden Markov models assume that the length of each region is geometrically distributed and the observed symbols are conditionally independently distributed. With a generalized hidden Markov model we can use different type of model to represent CGI and non-CGI regions, and also characterize the length of CGI regions with a distribution based on known data. In this section we show how we can use this ideas in ToPS to implement CGI characterization.

Our GHMM has only two states, shown in Figure **??**: CPG and $NONCPG$. We will model $NONCPG$ as a state with a geometric run-length distribution represented by a self transition, and the state CPG with an i.i.d. run-length distribution based on an histogram of known CPG island sizes. To characterize both CPG and NONCPG we will use Variable Length Markov Chains (VLMCs). VLMCs have the ability of representing dependencies of arbitrary length, and we hypothesize that this model can improve CPG detection.

Datasets

To characterize CpG islands and their lengths we used 1000 randomly chosen sequences from the CGI list of the UCSC Genome Browser [4]. To characterize non-CpG parts of a sequence we used 1000 randomly selected sequences of size 1000 from the human hg18 genome.

We used as a validation set the sequences from the hg18 assembly. We compared our results with two independentt CGI list: (i) CGI list obtained using HMM [3] stored as "Custom Annotation Tracks" in the UCSC Genome Browser; (ii) CGI list provided by UCSC Genome Browser [4]. We used the same method described in Glass and collaborators [5] to assess the quality of the CGI lists where the transcription start sites (TSS) of Refseq genes are used as references.

Building and training the models

To implement this system in ToPS we initially train the two VLMCs that will constitute the states of the GHMM. To do this we use the `train` program and the algorithm Context [6, 7]. We will run this algorithm for different values of the parameter (cut), beginning at value zero and ending at value 3, with 0.1 increments, and use BIC (Bayesian Information Criteria) [8] for model selection. This procedure is specified in the configuration files below, which also specify the input alphabet (nucleotides) and the location of the training sets:

`train_vlmc_cpg.txt`

```
training_algorithm= "ContextAlgorithm"
alphabet = ("A", "C", "G", "T")
model_selection_criteria="BIC"
begin = ("cut" : 0)
end = ("cut": 3)
step=("cut": 0.1)
training_set="dataset/cpg_hg18_random.fa"
```

`train_vlmc_notcpg.txt`

```
training_algorithm= "ContextAlgorithm"
alphabet = ("A", "C", "G", "T")
model_selection_criteria="BIC"
```

```
begin = ("cut" : 0)
end = ("cut": 3)
step=("cut": 0.1)
training_set="dataset/not_cpg_hg18_random.fa"
```

Running the train program will generate files with the VLMC models:

Command line
```
train -c cfg/train_vlmc_cpg.txt -F > model/cpg_vlmc.txt
```

Command line
```
train -c cfg/train_vlmc_notcpg.txt -F > model/not_cpg_vlmc.txt
```

We will also need to characterize the lengths of the CpG islands, to model the run length of the CPG state in our GHMM, . The configuration for the train program is very simple specifying only the training algorithm (Kernel Density [9]) and the training set:

train_cpg_duration.txt
```
training_algorithm= "SmoothedHistogramKernelDensity"
training_set="dataset/cpg_lengths.txt"
```

Running train produces the i.i.d. model:

Command line
```
train -c cfg/train_cpg_duration.txt > model/cpg_duration.txt
```

Once we have all the trained models, we specify the GHMM with another configuration file:

cpg_island_ghmm.txt
```
model_name = "GeneralizedHiddenMarkovModel"
observation_symbols = ("A", "C", "G", "T")
state_names = ( "NONCPG", "CPG")
initial_probabilities = ("CPG": 0.0; "NONCPG": 1)
terminal_probabilities = ("NONCPG": 0.5; "CPG": 0.5)
transitions = ("CPG" | "NONCPG": 0.0001;
               "NONCPG" | "NONCPG": 0.9999;
               "NONCPG" | "CPG": 1 )
cpg_duration = "cpg_duration.txt"
cpg_observation = "cpg_vlmc.txt"
noncpg_observation = "not_cpg_vlmc.txt"
# Description of the CPG and NONCPG states
CPG = [ duration="cpg_duration"
        observation = "cpg_observation"]
NONCPG = [ observation = "noncpg_observation"]
```

Evaluating the results

We can apply our model with the *viterbi_decoding* program to label the ENCODE regions. To assess the quality of our results we compared them with the UCSC genome browser CGI's and with the HMM's annotated CGI using the methodology proposed by Glass and collaborators [5], where the success of CGI prediction is measured by the rate of TSS regions covered by the CGI predictions.

Using other models

We have presented the use of ToPS to specify and train HMMs. In this section, we show how to specify and train the other available probabilistic models.

Independent Identically Distributed Model

We specify a discrete i.i.d. model using a vector of probabilities values. The file *fdd.txt* describes a distribution of two symbols: symbol *Sun* with probability 0.2, and symbol *Rain* with probability 0.8.

fdd.txt

```
model_name = "DiscreteIIDModel"
alphabet = ("Sun", "Rain")
probabilities = ( 0.2, 0.8)
```

Discrete i.i.d. model can also be used to represent histograms. In this case, the *alphabet* parameter is not necessary. We can estimate a smoothed histogram using the Kernel Density Estimation [9]. The file *histogram.txt* is an example of a configuration file for the *train* program.

histogram.txt

```
train_algorithm = "SmoothedHistogramKernelDensity"
training_set = sequences.txt
```

Variable length Markov chain

VLMCs are described by specifying the distribution associated with each context. The *vlmc.txt* file shows an example.

vlmc.txt

```
model_name = "VariableLengthMarkovChain"
alphabet = ("0", "1")
probabilities = ("0" | "": 0.5;
                 "1" | "": 0.5;
                 "0" | "1": 0.5;
                 "1" | "1": 0.5;
                 "0" | "0": 0.1;
                 "1" | "0": 0.9;
                 "0" | "1 0": 0.7;
                 "1" | "1 0": 0.3;
                 "0" | "1 1": 0.4;
                 "1" | "1 1": 0.6)
```

To estimate the probabilities of a VLMC, we can apply the Context algorithm [7]. The *contextAlgorithm.txt* file specifies the task of the program *train*.

contextAlgorithm.txt

```
training_algorithm = "ContextAlgorithm"
alphabet = ("0", "1")
cut = 0.5
training_set = "input.txt"
```

Inhomogeneous Markov model

To create a inhomogeneous Markov model, we have to specify the conditional probabilities for each position of the sequence. The file *ihm.txt* has an example of how we can specify this model.

ihm.txt

```
model_name = "InhomogeneousMarkovChain"
p1 = ("A" | "" : 0.97;
      "C" | "" : 0.01;
      "G" | "" : 0.01 ;
      "G" | "" : 0.01)
p2 = ("A" | "" : 0.01;
      "C" | "" : 0.97;
      "G" | "" : 0.01 ;
      "G" | "" : 0.01)
p3 = ("A" | "" : 0.01;
      "C" | "" : 0.01;
      "G" | "" : 0.97 ;
      "G" | "" : 0.01)
position_specific_distribution = ("p1","p2","p3")
phased =0
alphabet = ("A", "C", "G", "T")
```

The *position_specific_distribution* argument uses the parameters p1, p2, and p3 to specify respectively the distributions for the positions 1, 2, and 3 of the sequence.

The file *wam.txt* specifies a training procedure, described in [10], for an inhomogeneous Markov chain.

wam.txt

```
  training_algorithm = "WeightArrayModel"
  order = 2
  training_set = "train.txt"
  alphabet = ("A", "C", "G", "T")
  sequence_length = 10
  vicinity_length = 0
```

Using model selection when training a probabilistic model

Many models would have different dimensionality which are defined by the user during the training procedure. Typical example includes Markov chain models in which the user has to choose the value of the order. To help find the best set of such parameters. ToPS contains two model selection criteria that the user can use with a training algorithm.

- Bayesian Information Criteria (BIC) [8]: *This criteria selects the model with the largest value for the formula below:*

$$\log(Maximum\ Likelihood) - \frac{1}{2}(number\ of\ independently\ adjusted\ parameters) \times \log(sample\ size)$$

- Akaike Information Criteria (AIC) [11]: *This criteria selects the model with the smallest value for the formula:*

$$(-2)\log(Maximum\ Likelihood) + 2(number\ of\ independently\ adjusted\ parameters)$$

To run a model selection procedure the user have to specify four arguments:

- *model_selection_criteria* specifies the model selection criteria: BIC, or AIC.
- *begin* specifies the set of parameters to be tested and their initial values.
- *end* specifies the final values for the parameters specified above
- *step* specifies the increment on the values of each of the parameters being tested.

For example, the file `bic.txt` specifies that ToPS will use BIC selection criteria. The training procedure will calculate the BIC values for the estimated VLMC for each cut in the set $\{0.0, ..., 1.0\}$, and it will return the model with the preferred BIC value.

bic.txt

```
training_algorithm = "ContextAlgorithm"
training_set = "sequences.txt"
model_selection_criteria = "BIC"
begin = ("cut": 0.0)
end = ("cut": 1.0)
step = ("cut": 0.1)
alphabet = ("0", "1")
```

References

1. Durbin R, Eddy SR, Krogh A, Mitchison G (1998) Biological sequence analysis: Probabilistic models of proteins and nucleic acids. Cambridge University Press.

2. Rabiner LR (1989) A tutorial on Hidden Markov Models and selected applications in speech recognition. In: Proccedings of the IEEE. volume 77, pp. 257-286.

3. Wu HAO, Caffo B, Jaffee HA, Irizarry RA (2010) Redefining CpG islands using hidden Markov models 1: 499–514.

4. Kent WJ, Sugnet CW, Furey TS, Roskin KM, Pringle TH, et al. (2002) The Human Genome Browser at UCSC. Genome Research 12: 996–1006.

5. Glass JL, Thompson RF, Khulan B, Figueroa ME, Olivier EN, et al. (2007) CG dinucleotide clustering is a species-specific property of the genome. Nucleic acids research 35: 6798–807.

6. Rissanen J (1983) A universal data compression system. Information Theory, IEEE Transactions on 29: 656–664.

7. Galves A, Löcherbach E (2008) Stochastic chains with memory of variable length. arXiv math.PR.

8. Schwarz G (1978) Estimating the dimension of a model. The Annals of Statistics 6: 461–464.

9. Sheather S (2004) Density estimation. Statistical Science 19: 588–597.

10. Burge C (1997) Identification of genes in human genomic DNA. Ph.D. thesis, Stanford University.

11. Akaike H (1974) A new look at the statistical model identification. IEEE transactions on automatic control AC-19: 716-723.
