# Supplementary material for: ToPS: A Framework to Manipulate Probabilistic Models of Sequence Data
Source: PLoS Comput Biol. 2013 Oct 3;9(10):e1003234. doi: 10.1371/journal.pcbi.1003234 (PMC3789777; doi:10.1371/journal.pcbi.1003234)
Supplement: Software S1 — Source code for ToPS. A compressed file containing the source code for ToPS. (GZ) [file pcbi.1003234.s001.gz › tops/doc/manual/diagrama_classe.pdf]

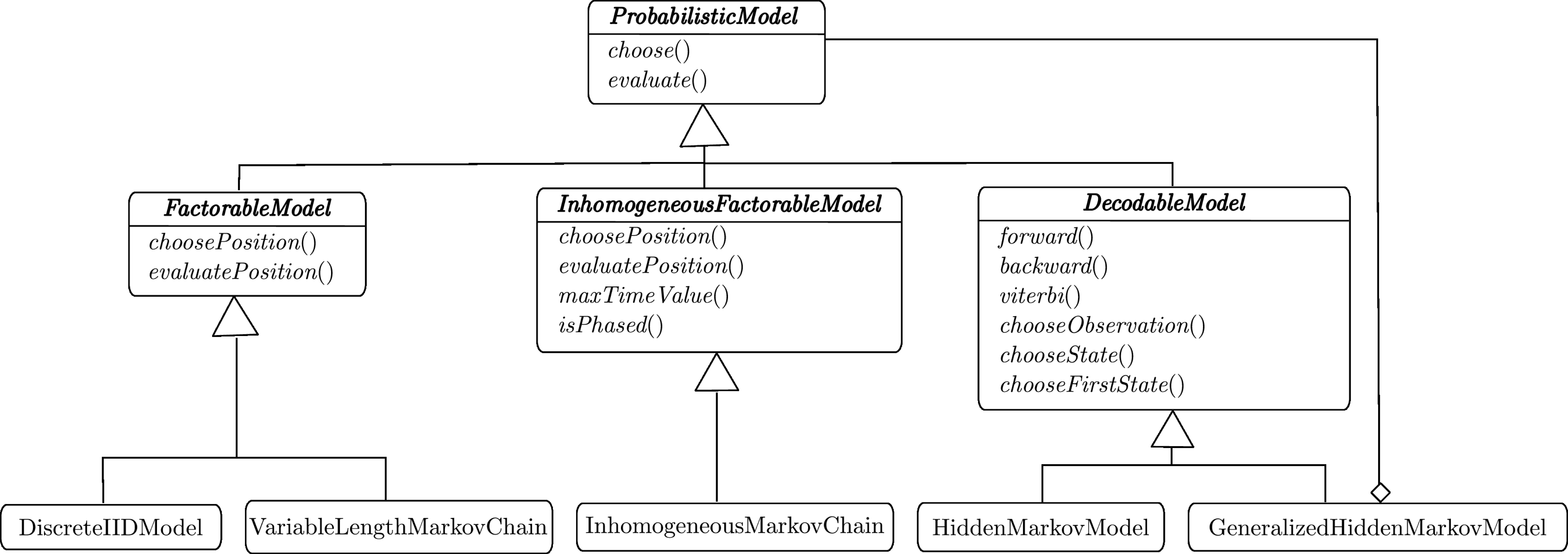
ProbabilisticModel
choose()
evaluate()
FactorableModel
choosePosition()
evaluatePosition()
InhomogeneousFactorableModel
choosePosition()
evaluatePosition()
maxTimeValue()
isPhased()
DecodableModel
forward()
backward()
viterbi()
chooseObservation()
chooseState()
chooseFirstState()
DiscreteIIDModel
VariableLengthMarkovChain
InhomogeneousMarkovChain
HiddenMarkovModel
GeneralizedHiddenMarkovModel
