# Supplementary material for: ToPS: A Framework to Manipulate Probabilistic Models of Sequence Data
Source: PLoS Comput Biol. 2013 Oct 3;9(10):e1003234. doi: 10.1371/journal.pcbi.1003234 (PMC3789777; doi:10.1371/journal.pcbi.1003234)
Supplement: Software S1 — Source code for ToPS. A compressed file containing the source code for ToPS. (GZ) [file pcbi.1003234.s001.gz › tops/doc/manual/fig_cassino.pdf]

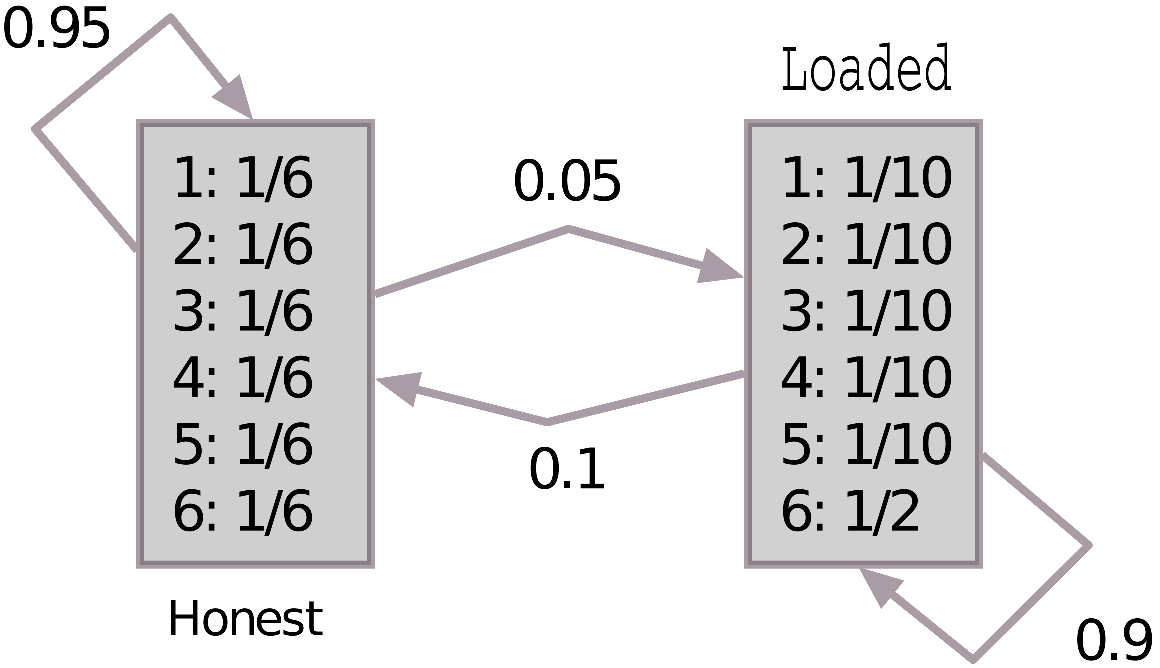
0.95
Loaded
1: 1/6
2: 1/6
3: 1/6
4: 1/6
5: 1/6
6: 1/6
0.05
1: 1/10
2: 1/10
3: 1/10
4: 1/10
5: 1/10
6: 1/2
0.1
Honest
0.9
