# Supplementary material for: ToPS: A Framework to Manipulate Probabilistic Models of Sequence Data
Source: PLoS Comput Biol. 2013 Oct 3;9(10):e1003234. doi: 10.1371/journal.pcbi.1003234 (PMC3789777; doi:10.1371/journal.pcbi.1003234)
Supplement: Software S1 — Source code for ToPS. A compressed file containing the source code for ToPS. (GZ) [file pcbi.1003234.s001.gz › tops/doc/manual/tops-doc.pdf]

ToPS User Guide

André Yoshiaki Kashiwabara
Ígor Bonadio
Vitor Onuchic
Alan Mitchell Durham

January de 2013

Contents

1 Introduction **1**

1.1 Supported Features 1

2 Build and Installation **3**

2.1 Requirements 3

2.2 Building from Source 3

3 Sequence Formats **5**

3.1 FASTA format 5

3.2 ToPS sequence format 5

4 Describing Probabilistic Models **7**

4.1 Independent Identically Distributed Model 7

4.2 Variable Length Markov Chain 8

4.3 Hidden Markov Model 8

4.4 Inhomogeneous Markov Model 9

4.5 Pair Hidden Markov Model 10

4.6 Profile Hidden Markov Model 11

4.7 Generalized Hidden Markov Model 13

4.8 Language Description (EBNF) 15

5 Training Probabilistic Models **17**

5.1 The *train* program 18

5.2 Discrete IID Model 18

5.3 Context Algorithm 18

5.4 Fixed Length-Markov Chain 18

5.5 Training the GHMM transition probabilities 19

5.6 Training HMM using algorithm Baum-Welch 19

5.7 Interpolated Markov Chain 19

5.8 Profile HMM Maximum Likelihood 19

5.9 Profile HMM Baum-Welch 20

5.10 Weight Array Model 20

5.11 Phased Markov Model 20
5.12 Smoothed Histogram (Burge) 21
5.13 Smoothed Histogram (Stanke) 21
5.14 Smoothe Histogram (Kernel Density Estimation) 21
5.15 Similarity Based Sequence Weighting 21
5.16 Using model selection when training a probabilistic model 22

6 Simulating Probabilistic Models **23**

7 Evaluating probabilities of a sequence **25**

8 Other Applications **27**
8.1 Aligning Using Pair HMM 27
8.2 Bayesing Classifier 27
8.3 Sliding Window 28
8.4 Viterbi Decoding and Posterior Decoding 28

9 Design and Implementation **29**
9.1 ProbabilisticModel hierarchy 29
9.1.1 FactorableModel 30
9.1.2 InhomogeneousFactorableModel 31
9.1.3 DecodableModel 31
9.2 ProbabilisticModelCreator and ProbabilisticModelParameterValue hierarchies 32

Bibliography **33**

Chapter 1

Introduction

Probabilistic models for sequence data play an important role in many dicipline such as natural language processing [MS99], computational music [KD06], and Bioinformatics [DEKM98]. Examples of such models include hidden Markov models [Rab89], hidden semi-Markov model [Gué03] also know as Generalized Hidden Markov Model [KHRE96, Bur97], and variable-length Markov chain [Ris83].

This document describes the usage of ToPS (Toolkit of Probabilistic Model of Sequence) that combines in a single environment the mechanisms to manipulate different probabilistic models. Currently, ToPS contains the implementation of the following models:

1. Independent and identically distributed model
2. Variable-Length Markov Chain (VLMC)
3. Inhomogeneous Markov Chain
4. Hidden Markov Model
5. Pair Hidden Markov Model
6. Profile Hidden Markov Model
7. Generalized Hidden Markov Model (GHMM)

The user can implement models either by manual description of the probability values in a configuration file, or by using training algorithms provided by the system. The ToPS framework also includes a set of programs that implement bayesian classifiers, sequence samplers, and sequence decoders. Finally, ToPS is an extensible and portable system that facilitates the implementation of other probabilistic models, and the development of new programs.

1.1 Supported Features

1. ToPS contains a simple language near to the mathematical notation that can be used to describe the parameters of different type of models.
2. ToPS allows the use of any implemented model to represent the emissions of the GHMM's states.
3. Sequence samplers are available.

4. ToPS contains the implementation of Viterbi decoding, forward and backward algorithmhs for "decodable" models (HMM, pair-HMM, profile-HMM, and GHMM).
5. Baum-Welch training is implemented for HMM, and pair-HMM.
6. Maximum Likelihood training (profile-HMM, and Markov chains)
7. The object-oriented design of ToPS is extensible and developers are welcome to include the implementation of other probabilistic models or algorithms.
8. The ToPS source-code is under the GiT version control system (http://git-scm.com).
9. ToPS provides the implementation of many distinct and different programs:
   - aligner
   - sequence sampler
   - bayesian classifier
   - sliding window analysis
   - posterior decoding
   - viterbi decoding
   - path sampler given a sequence and a GHMM

Chapter 2

Build and Installation

This chapter provides a complete guide to building and installing ToPS.

2.1 Requirements

ToPS was designed to run on Unix/Linux operating systems, but it should work on Windows too. Tested platforms include: MacOS X and Ubuntu linux.

This framework was written in C++ and its requirements are listed below:

* *G++ v4.2.1* - http://gcc.gnu.org/
* *Boost C++ v1.52* - http://www.boost.org/
* *CMake v2.8.8* - http://www.cmake.org/
* *Git v1.7.9* - http://git-scm.com/
* *GoogleTest v1.5.0* - http://code.google.com/p/googletest/

2.2 Building from Source

1. Download the last version of ToPS using Git

   ```
   git clone git://tops.git.sourceforge.net/gitroot/tops/tops
   ```

   This will create a directory named tops

2. Install the google test framework submodule.

   ```
   git submodule update --init
   ```

3. Go to the tops directory:

   ```
   cd tops
   ```

4. Run the configuration script:

   ```
   cmake .
   ```

5. Run make and make install

```
make
sudo make install
```

Chapter 3

Sequence Formats

ToPS can read two distinct text-based file format: (1) FASTA; (2) ToPS sequence format.

3.1 FASTA format

FASTA format has become a standard in the field of Bioinformatics and it represents any sequence data such as nucleotide sequences or protein sequences.

A sequence in FASTA format always begins with a single-line description, followed by the lines of the sequence data. The description line begins with the greater-than (">") symbol. The sequence data ends when another ">" appears or when the end-of-file is encountered.

Example:

```
>chr13_72254614
AGGAGAGAATTTGTCTTTGAATTCACTTTTTCTTACCTATTTCCC
TTCAAAAAGAAGGAGGGAGGCCGATCTTAGTATAGTTCTCGCTGT
TTCCCCTCCACACACCTTTCCTTATCATTCAGTTTAGAAAAACTG
AAATATTTAATAGCATAATTTGTTATATCATGAGGTATTAAAACA
AGGTAGTTGCTAACATTCTTATGAGAGAGTTAGAAGTAAGTTCTA
>chr12_54396566
ACTCTGGAGGGAGGAGGGTGTGGGGAACCCCCCAGAGATGGGCTT
CTTGGAGGCCTGAAACCACCGGAACGGAGGTGGGGCACTTGTTTC
CTGAGTCCGGGCTGGAAATCTCGGAGTTACCGATTCTGCGGCCGA
GTAGTGGAGAAAGAGTGCCTGGGAGTCAGGAGTCCTGGGCGCTGC
CGCTGACTTCCTGGCGTCCCTGAGTGAGTCCATTTCCCTCCCAGG
```

3.2 ToPS sequence format

ToPS sequence format assumes that each line contains a single sequence data. A sequence in ToPS sequence format begins with a description followed by the two-dots (":") symbol, followed by a space, followed by the sequence data. ToPS allows the presence of multiple-character symbols, and the symbols in the sequence data are isolated by a space.

Example:

```
chr13_72254614: A G G A G A G A A T T T G T C T T T
seq1234: CPG CPG NOCPG CPG CPG CPG CPG NOCPG
```

Chapter 4

Describing Probabilistic Models

ToPS uses its own language to describe models and configurations. This is a simple language that will help users without previous knowledge in programming to define the parameters of the model and to do sequence analysis experiments.

To use a model, you need to define a mandatory parameter called "model_name" to specify which model you will use. Currently, available "model_name" values are:

- `DiscreteIIDModel`
- `VariableLengthMarkovChain`
- `HiddenMarkovModel`
- `InhomogeneousMarkovChain`
- `PairHiddenMarkovModel`
- `ProfileHiddenMarkovModel`
- `GeneralizedHiddenMarkovModel`

In this chapter we describe how the user can define the specific parameters of each model using simple examples. Finally, we show the formal specification of the language in the Extended Backus-Naur Form (EBNF).

4.1 Independent Identically Distributed Model

We specify a discrete i.i.d. model using a vector of probabilities values. The file *fdd.txt* describes a distribution of two symbols: symbol *Sun* with probability 0.2, and symbol *Rain* with probability 0.8.

fdd.txt

```
model_name = "DiscreteIIDModel"
alphabet = ("Sun", "Rain")
probabilities = (0.2, 0.8)
```

When the "alphabet" is not present, it means that we are describing the distribution over non-negative integers numbers. For example, the file described below is specifying that the probability of 0 is 0.2, the probability of 1 is 0.1, the probability of 2 is 0.3 and the probability of 3 is 0.4.

```
model_name = "DiscreteIIDModel"
probabilities = (0.2, 0.1, 0.3, 0.4)
```

4.2 Variable Length Markov Chain

VLMCs are described by specifying the distribution associated with each context. The *vlmc.txt* file shows an example. We use the `probabilities` parameter to specify the conditional probabilities, for example, line 9 specifies that the probability of $X_n = 0$ given that $X_{n-1} = 1$ and $X_{n-2} = 2$ is 0.7.

vlmc.txt

```
1  model_name = "VariableLengthMarkovChain"
2  alphabet = ("0", "1")
3  probabilities = (
4    "0" | "": 0.5;
5    "1" | "": 0.5;
6    "0" | "1": 0.5;
7    "1" | "1": 0.5;
8    "0" | "0": 0.1;
9    "1" | "0": 0.9;
10   "0" | "1 0": 0.7;#P(X_n=0|X_{n-1}=1,X_{n-2}=0)=0.7
11   "1" | "1 0": 0.3;
12   "0" | "1 1": 0.4;
13   "1" | "1 1": 0.6)
```

4.3 Hidden Markov Model

A simple example where HMM can be used is in the dishonest casino problem. A dishonest casino has two different dice, one is loaded and the other is fair. The casino can change the die without the player knowing and the challenge is to predict when the casino has changed the dice. Figure 4.1 shows the HMM for this problem. This model has two states (Fair, and Loaded). When the model is in Loaded state there is a greater probability to observe the number one than the other numbers, and when the model is in Fair state the numbers are uniformly distributed. The file below shows an example of HMM described using ToPS:

"hmm.txt"

```
# Dishonest Casino Problem
model_name="HiddenMarkovModel"
state_names= ("Fair", "Loaded" )
observation_symbols= ("1", "2", "3", "4", "5", "6" )
# transition probabilities
transitions = ("Loaded"    | "Fair": 0.1;
               "Fair"      | "Fair": 0.9;
               "Fair"      | "Loaded": 0.1;
               "Loaded"    | "Loaded": 0.9 )
# emission probabilities
emission_probabilities = ("1" | "Fair" : 0.166666666666;
                          "2" | "Fair" : 0.166666666666;
                          "3" | "Fair" : 0.166666666666;
                          "4" | "Fair" : 0.166666666666;
                          "5" | "Fair" : 0.166666666666;
                          "6" | "Fair" : 0.166666666666;
                          "1" | "Loaded" : 0.5;
                          "2" | "Loaded" : 0.1;
```

```
                              "3" | "Loaded" : 0.1;
                              "4" | "Loaded" : 0.1;
                              "5" | "Loaded" : 0.1;
                              "6" | "Loaded" : 0.1)
initial_probabilities= ("Fair": 0.5; "Loaded": 0.5)
```

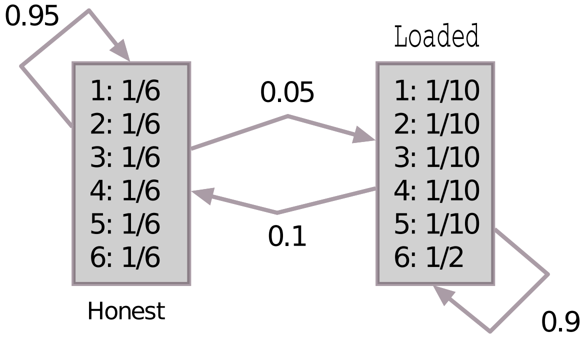

Figure 4.1: *Dishonest Casino Problem*

4.4 Inhomogeneous Markov Model

To create an inhomogeneous Markov model, we have to specify the conditional probabilities for each position of the sequence. The file *ihm.txt* has an example of how we can specify this model.

ihm.txt

```
model_name = "InhomogeneousMarkovChain"
p1 = ("A" | "" : 0.97;
      "C" | "" : 0.01;
      "G" | "" : 0.01 ;
      "G" | "" : 0.01)
p2 = ("A" | "" : 0.01;
      "C" | "" : 0.97;
      "G" | "" : 0.01 ;
      "G" | "" : 0.01)
p3 = ("A" | "" : 0.01;
      "C" | "" : 0.01;
      "G" | "" : 0.97 ;
      "G" | "" : 0.01)
position_specific_distribution = ("p1","p2","p3")
phased =0
alphabet = ("A", "C", "G", "T")
```

The *position_ specific_ distribution* argument uses the parameters p1, p2, and p3 to specify respectively the distributions for the positions 1, 2, and 3 of the sequence.

In this example the *phased* parameter, with value equals to zero, is specifying that the model is describing fixed-length sequences. A model that represents fixed-length sequences is useful when we want to model biological signal. Weight Array Model [ZM93] is an example of this type of Inhomogeneous Markov Chain.

If the *phased* is equal to one, then the sequences are generated using periodically the distribution p1, p2, and p3. This behaviour is useful to model coding regions of the gene. Three-periodic Markov chain [BM93] is an example of a inhomogeneous Markov Chain with phased equals to 1 and three position specific distributions.

4.5 Pair Hidden Markov Model

A very common problem when analyzing biological sequences is that of aligning a pair of sequences. This task can be done through the use of decodable models, although in this case these models must be able to handle a pair of sequences simultaneously. Here we define this kind of model a pair hidden Markov model (PHMM). This pairHMM has a Match state (M), two insertion states (I1, I2), two deletion state (D1, D2) an initial state (B), and a final state (E).

ihm.txt

```
model_name="PairHiddenMarkovModel"
state_names = ("M", "I1", "D1", "I2", "D2", "B", "E")
observation_symbols = ("A","C","G","T")
transitions = ("M" | "B" :0.9615409374;
                "I1" | "B" : 4.537999985e-07;
                "D1" | "B" : 4.537999985e-07;
                "I2" | "B" : 0.01922916807;
                "D2" | "B" : 0.01922916807;
                "I1" | "M" : 0.01075110921;
                "D1" | "M" : 0.01075110921;
                "I2" | "M" : 0.008213998383;
                "D2" | "M" : 0.008213998383;
                "M" | "M" : 0.9619031182;
                "I1" | "I1" : 0.3209627509;
                "D1" | "D1" : 0.3209627509;
                "I2" | "I2" : 0.3297395944;
                "D2" | "D2" : 0.3297395944;
                "M" | "I1" : 0.6788705825;
                "M" | "D1" : 0.6788705825;
                "M" | "I2" : 0.670093739;
                "M" | "D2" : 0.670093739;
                "E" | "M" : 0.000166667;
                "E" | "I1" : 0.000166667;
                "E" | "D1" : 0.000166667;
                "E" | "I2" : 0.000166667;
                "E" | "D2" : 0.000166667;)
emission_probabilities = ("AA" | "M" : 0.1487240046;
                        "AT" | "M" : 0.0238473993;
                        "AC" | "M" : 0.0184142999;
                        "AG" | "M" : 0.0361397006;
```

```
                       "TA" | "M" : 0.0238473993;
                       "TT" | "M" : 0.1557479948;
                       "TC" | "M" : 0.0389291011;
                       "TG" | "M" : 0.0244289003;
                       "CA" | "M" : 0.0184142999;
                       "CT" | "M" : 0.0389291011;
                       "CC" | "M" : 0.1583919972;
                       "CG" | "M" : 0.0275536999;
                       "GA" | "M" : 0.0361397006;
                       "GT" | "M" : 0.0244289003;
                       "GC" | "M" : 0.0275536999;
                       "GG" | "M" : 0.1979320049;
                       "A-" | "I1" : 0.2270790040;
                       "T-" | "I1" : 0.2464679927;
                       "C-" | "I1" : 0.2422080040;
                       "G-" | "I1" : 0.2839320004;
                       "-A" | "D1" : 0.2270790040;
                       "-T" | "D1" : 0.2464679927;
                       "-C" | "D1" : 0.2422080040;
                       "-G" | "D1" : 0.2839320004;
                       "A-" | "I2" : 0.2270790040;
                       "T-" | "I2" : 0.2464679927;
                       "C-" | "I2" : 0.2422080040;
                       "G-" | "I2" : 0.2839320004;
                       "-A" | "D2" : 0.2270790040;
                       "-T" | "D2" : 0.2464679927;
                       "-C" | "D2" : 0.2422080040;
                       "-G" | "D2" : 0.2839320004;)
number_of_emissions = ("M" : "1,1";
                       "I1" : "1,0";
                       "D1" : "0,1";
                       "I2" : "1,0";
                       "D2" : "0,1";
                       "B" : "0,0";
                       "E" : "0,0")
```

4.6 Profile Hidden Markov Model

Biologial sequences usually come in families and a very common problem when analyzing this sequences is identify the relationship of an individual sequence to a sequence family. Profile HMMs turn a multiple sequence alignment into a position-specific scoring system on which is possible to perform a database search for more members. This kind of model is described in the *profilehmm.txt* file and has five match states $M0, M1, M2, M3, M4$ ($M0$ and $M4$ are modeled as the begin and end states respectively), four insert states $I0, I1, I2, I3$ and three delete states $D1, D2, D3$.

profilehmm.txt
```
model_name = "ProfileHiddenMarkovModel"
state_names = ("M0","M1","M2","M3","M4","I0","I1","I2","I3",
               "D1","D2","D3")
observation_symbols = ("A","C","G","T")
transitions = ("M1" | "M0": 0.625;
               "I0" | "M0": 0.125;
               "D1" | "M0": 0.25;
               "M2" | "M1": 0.714286;
               "I1" | "M1": 0.142857;
               "D2" | "M1": 0.142857;
               "M3" | "M2": 0.428571;
               "I2" | "M2": 0.428571;
               "D3" | "M2": 0.142857;
               "M4" | "M3": 0.833333;
               "I3" | "M3": 0.166667;
               "M4" | "M4": 1;
               "M1" | "I0": 0.333333;
               "I0" | "I0": 0.333333;
               "D1" | "I0": 0.333333;
               "M2" | "I1": 0.333333;
               "I1" | "I1": 0.333333;
               "D2" | "I1": 0.333333;
               "M3" | "I2": 0.272727;
               "I2" | "I2": 0.545455;
               "D3" | "I2": 0.181818;
               "M4" | "I3": 0.5;
               "I3" | "I3": 0.5;
               "M2" | "D1": 0.25;
               "I1" | "D1": 0.25;
               "D2" | "D1": 0.5;
               "M3" | "D2": 0.25;
               "I2" | "D2": 0.5;
               "D3" | "D2": 0.25;
               "M4" | "D3": 0.666667;
               "I3" | "D3": 0.333333)
emission_probabilities = ("A" | "M1": 0.5;
               "C" | "M1": 0.125;
               "G" | "M1": 0.25;
               "T" | "M1": 0.125;
               "A" | "M2": 0.25;
               "C" | "M2": 0.125;
               "G" | "M2": 0.5;
               "T" | "M2": 0.125;
               "A" | "M3": 0.125;
               "C" | "M3": 0.625;
               "G" | "M3": 0.125;
               "T" | "M3": 0.125;
               "A" | "I0": 0.25;
```

```
                    "C" | "I0": 0.25;
                    "G" | "I0": 0.25;
                    "T" | "I0": 0.25;
                    "A" | "I1": 0.25;
                    "C" | "I1": 0.25;
                    "G" | "I1": 0.25;
                    "T" | "I1": 0.25;
                    "A" | "I2": 0.5;
                    "C" | "I2": 0.25;
                    "G" | "I2": 0.166667;
                    "T" | "I2": 0.0833333;
                    "A" | "I3": 0.25;
                    "C" | "I3": 0.25;
                    "G" | "I3": 0.25;
                    "T" | "I3": 0.25)
initial_probabilities = ("M0": 1)
```

4.7 Generalized Hidden Markov Model

GHMMs are useful in Bioinformatics to represent the structure of genes. As an illustrative example we will use a simplified model for a bacterial gene. In bacteria, genes are regions of the genome with a different composition, specific start and stop signals, and noncoding regions separating different genes. Figure 4.2 illustrates this gene model. The model has four states: *NonCoding* state, representing the intergenic regions, with geometric duration distribution (represented by a self transition in the figure); *Start* and *Stop* states , representing the signals at the boundaries of a gene, with a fixed duration distribution ; *Coding*, representing the coding region of a gene, with an i.i.d. duration distribution. Box *ghmm.txt* shows the description of this GHMM.

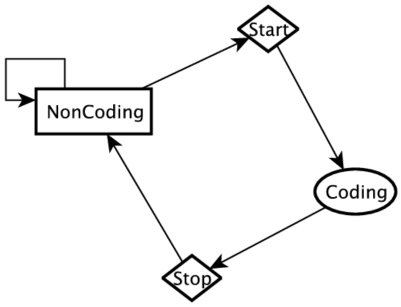

Figure 4.2: *GHMM that represents protein-coding genes in bacteria.*

The parameters *state_names*, *observation_symbols*, *initial_probabilities*, and *transitions* are configured in the same way as in the case of the HMM model, described above.

We have to specify the models that the GHMM will use, either by naming a file that contains its description or by inlining its description in the GHMM specification file. In our example, the GHMM uses five submodels: (i) *noncoding_model* (a *DiscreteIIDModel* inlined in the GHMM specification); (ii) *coding_model* (in file “coding.txt”) ; (iii) *start_model* (

in file “start.txt”); (iv) *stop_ model* (in file “stop.txt”); (v) *coding_ duration_ model* (in file “coding_duration.txt”).

After specifying the models, we have to describe the configuration of each state. ToPS assumes that the GHMM has two classes of states: (i) Fixed-length states, that emit fixed length words, and (ii) variable-length states, that emit words with lengths given by a probability distribution. There are two types of variable-length states: states with geometric distributed duration and states with non-geometric distributed duration. When specifying any state, the user have to specify the observation model using the parameter *observation*. States with geometric duration distribution are specified with a self transition, states with fixed-length dueation the user should use the parameter *sequence_ length*, and other states should use the parameter *duration*.

In the file *ghmm.txt*, we have two fixed-length states (*Start*, and *Stop*) and two variable-length states (*NonCoding*, and *Coding*):

- *Start* state, with *start_ model* as the observation model.
- *Stop* state, with *stop_ model* as the observation model.
- *NonCoding* state, with *noncoding_ model* as the observation model, and durations given by a geometric distribution in which the probability of staying in the same state is 0.999.
- *Coding* state, with *coding_ model* as the observation model, and durations given by the *coding_ duration_ model*.

ghmm.txt

```
model_name = "GeneralizedHiddenMarkovModel"
state_names =
  ("NonCoding",
   "Start",
   "Coding",
   "Stop")
observation_symbols =
  ("A", "C", "G", "T")
initial_probabilities =
  ("NonCoding": 1.0)
transitions =
  ("NonCoding"  | "NonCoding": 0.999;
        "Start" | "NonCoding": 0.001;
       "Coding" | "Start": 1.0;
         "Stop" | "Coding": 1.0;
   "NonCoding"  | "Stop": 1.0)
noncoding_model =
  [ model_name = "DiscreteIIDModel"
    alphabet = ("A", "C", "G", "T")
    probabilities=(0.25, 0.25, 0.25, 0.25)]
coding_model = "coding.txt"
start_model = "start.txt"
stop_model = "stop.txt"
coding_duration_model="coding_duration.txt"
NonCoding =
  [ observation = noncoding_model ]
```

```
Start =
  [ observation =start_model
    sequence_length = 15 ]
Stop =
  [ observation = stop_model
    sequence_length = 15 ]
Coding =
  [ observation = coding_model
    duration = coding_duration_model ]
```

4.8 Language Description (EBNF)

The configuration file of ToPS contains a list of defined properties. Each property is associated with a value that can be a string, integer, float number, a list of string, a list of numbers, conditional probabilities, or a list of other properties. Below, we describe the formal language description of ToPS in the Extended Backus-Naur Form (EBNF):

```
model                         : properties
                              ;

properties                    : property
                              | properties property
                              ;

property                      : IDENTIFIER '=' value
                              ;

value                         : STRING
                              | INTEGER_NUMBER
                              | FLOAT_POINT_NUMBER
                              | list
                              | probability_map
                              | conditional_probability_map
                              | sub_model
                              | IDENTIFIER
                              ;

list                          : '(' list_elements ')'
                              ;

list_elements                 : list_element
                              | list_elements ',' list_element
                              ;

list_element                  : STRING
                              | INTEGER_NUMBER
                              | FLOAT_POINT_NUMBER
                              ;

probability_map               : '(' probabilities_list ')'
                              ;

probabilities_list            : probabilities
                              | probabilities ';'
                              ;
```

```

probabilities                     : probability
                                  | probabilities ';' probability
                                  ;

probability                       : STRING ':' list_element
                                  ;

conditional_probability_map       : '(' conditional_probabilities_list ')'
                                  ;

conditional_probabilities_list    : conditional_probabilities
                                  | conditional_probabilities ';'
                                  ;

conditional_probabilities         : conditional_probability
                                  | conditional_probabilities ';'
                                      conditional_probability
                                  ;

conditional_probability           : condition ':' probability_number
                                  ;

condition                         : STRING '|' STRING
                                  ;

probability_number                : INTEGER_NUMBER
                                  | FLOAT_POINT_NUMBER
                                  ;

sub_model                         : '[' properties ']'
                                  ;
```

And the tokens are defined by the following regular expressions:

```
IDENTIFIER          : [a-zA-Z_][a-zA-Z0-9_]*
STRING              : L?\"(\\.|[^\\"])*\"
COMMENTS            : "#"[^\r\n]*
FLOAT_POINT_NUMBER  : [0-9]+\.[0-9]+([Ee][+-]?[0-9]+)?
INTEGER_NUMBER      : [0-9]+
```

Chapter 5

Training Probabilistic Models

To train a probabilistic model using ToPS, you need to create a file that will contain the parameters of the training procedure. In this file you have to specify the mandatory parameter "training_algorithm" that indicates the algorithm to be used to estimate the parameters of the model. Currently, the following "training_algorithm" values are available:

- `ContextAlgorithm`
- `DiscreteIID`
- `WeightArrayModel`
- `GHMMTransition`
- `FixedLengthMarkovChain`
- `BaumWelchHMM`
- `MaximumLikelihoodHMM`
- `PHMMBaumWelch`
- `ProfileHMMMaxLikelihood`
- `ProfileHMMBaumWelch`
- `PhasedMarkovChain`
- `InterpolatedMarkovChain`
- `SmoothedHistogramKernelDensity`
- `SmoothedHistogramKernelStanke`
- `SmoothedHistogramKernelBurge`

In this chapter we describe how the user can use each training algorithm.

5.1 The *train* program

ToPS provides a program, called "train", that receives a file with the parameters of a training procedure and returns the model description to the standard output. The command line below is an example of how you can run the program.

Command line
```
train -c  training_specification.txt > model.txt
```

The command line parameter of the *train* program is:

- -c specifies the name of the file containing the training parameters

5.2 Discrete IID Model

To train discrete i.i.d. model, you need to specify the training set and an alphabet. The *DiscreteIID* algorithm estimates the probability of each symbol using maximum likelihood method and it returns the *DiscreteIIDModel* specification.

trainiid.txt
```
training_algorithm="DiscreteIIDModel"
alphabet = ("A", "C", "G", "T")
training_set="sequence_from_discreteiid.txt"
```

5.3 Context Algorithm

To train variable length Markov chains, you can use the algorithm Context [Ris83, GL08]. It receives a training set, the alphabet, and the parameter *cut*. The *cut* specifies a threshold for the pruning of the probabilistic suffix tree. The greater the value of the *cut*, the smaller will be the tree, because more nodes will be considered indistinguishable with their descendents. The output of this algorithm is a "VariableLengthMarkovChain" description.

bic.txt
```
training_algorithm = "ContextAlgorithm"
training_set = "sequences.txt"
cut = 0.1
alphabet = ("0", "1")
```

5.4 Fixed Length-Markov Chain

To train a fixed order Markov chain, you can use the algorithm `FixedLengthMarkovChain`. It receives a training set, the alphabet, and the parameter "order". The output of this algorithm is a "VariableLengthMarkovChain" specification where the contexts have length equals to the value specified using the *order* parameter. The file *fdd.txt* is describing the training of a Markov chain of order 1.

fdd.txt
```
training_algorithm = "FixedLengthMarkovChain"
training_set = "sequences.txt"
order = 1
alphabet = ("0", "1")
```

5.5 Training the GHMM transition probabilities

To estimate the transition probabilities of a GHMM using maximum likelihood method, you can use the algorithm `GHMMTransition`. It receives a training set containing the a set of sequences of states and an initial ghmm model. It returns a new GHMM with the estimated transition probabilities.

train_transitions.txt
```
training_algorithm="GHMMTransitions"
training_set="train.txt"
ghmm_model="ghmm.txt"
```

5.6 Training HMM using algorithm Baum-Welch

To estimate the parameter of a HMM, given an initial HMM, you can use the algorithm *BaumWelchHMM*. It receives a training set, an initial HMM model, and the maximum number of iteration. It returns a new *HiddenMarkovModel* with the estimated parameters.

train.txt
```
training_algorithm = "BaumWelchHMM"
training_set = "trainhmm_bw.sequences"
initial_specification = "initial_hmm.txt"
maxiter=300
```

5.7 Interpolated Markov Chain

To train the parameters of an interpolated Markov chain [SDKW98], you can use the *InterpolatedMarkovChain* training algorithm. It receiveis a training set, the order of the Markov chain, and it will return a *VariableMarkovChain* specification with the estimated parameters.

train.txt
```
training_algorithm="InterpolatedMarkovChain"
training_set="intergenic.fasta"
alphabet=( "A", "C", "G", "T" )
pseudo_counts=1
order=4
```

5.8 Profile HMM Maximum Likelihood

To estimate the parameters of a Profile HMM, you can use de *ProfileHMMMaxLikelihood* algorithm. It receives an alignment in the fasta format file, the alphabet of the model, the

residue fraction which is used to decide what multiple alignment columns assign as match, insert and delete states, and the pseudocounts value. It returns a *Profile HMM* with the estimated parameters.

train.txt
```
training_algorithm="ProfileHMMMaxLikelihood"
fasta_file="fasta_alignment.txt"
alphabet="A,C,G,T"
residue_fraction=0.7
pseudocounts=1
```

5.9 Profile HMM Baum-Welch

You can also use de *ProfileHMMBaumWelch* algorithm to estimate the parameters of a profile. It receives an initial Profile HMM model, the training set that should be formated in the ToPS sequence format file, the threshold, the maximum number of iterations, and the pseudocounts value. It returns a new *Profile HMM* with the estimated parameters.

train.txt
```
training_algorithm="ProfileHMMBaumWelch"
initial_model="profile.txt"
training_set="training_sequences.txt"
threshold = 0.03
maxiter= 500
pseudocounts = 1
```

5.10 Weight Array Model

Weight array models [Bur97, ZM93] are inhomogeneous Markov models that represent fixed-length sequences. It is useful to model biological signal, such as splicing sites, branch points sites, and stop codons. To train a WAM, you can use the *WeightArrayModel* algorithm, it receives a set of sequences with length specified using the *length* parameter. This training algorithm returns a *InhomogeneousMarkovChain* description with the estimated parameters.

train.txt
```
training_algorithm = "WeightArrayModel"
training_set="sequences.txt"
length=3
alphabet=("0", "1")
order=1
```

5.11 Phased Markov Model

Phased Markov Model is an Inhomogeneous Markov Model for which the positional distributions are periodically reused to generate the sequences. Three-periodic Markov Model [BM93] is an example of this model and it is useful to represent protein-coding regions of the gene.

train.txt
```
training_algorithm="PhasedMarkovChain"
training_set="sequence.fasta"
```

```
alphabet=("A", "C", "G", "T")
order=4
pseudo_counts = 0
number_of_phases=3
phased = 1
```

5.12 Smoothed Histogram (Burge)

To create a smoothed histogram of positive integers, you can use the *SmoothedHistogram-Burge* [Bur97]. It receives a training set containing a sequence of positive integers and it returns a *DiscreteIIDModel* with the estimated probabilities of for each number.

train.txt

```
training_algorithm = "SmoothedHistogramBurge"
training_set = "lengths.txt"
C=1.0
```

5.13 Smoothed Histogram (Stanke)

To create a smoothed histogram of positive integers, you can use the *SmoothedHistogram-Stanke* [Sta03]. It receives a training set containing a sequence of positive integers and it returns a *DiscreteIIDModel* with the estimated probabilities of for each number.

train.txt

```
training_algorithm = "SmoothedHistogramStanke"
training_set = "lengths.txt"
```

5.14 Smoothe Histogram (Kernel Density Estimation)

To create a smoothed histogram of positive integers, you can use the *SmoothedHistogramKernelDensity* [She04]. It receives a training set containing a sequence of positive integers and it returns a *DiscreteIIDModel* with the estimated probabilities of for each number.

train.txt

```
training_algorithm = "SmoothedHistogramKernelDensity"
training_set = "lengths.txt"
```

5.15 Similarity Based Sequence Weighting

To create a Similarity Based Sequence Weighting [SW03], you can use the SBSW training algorithm. It receives a training set containing fixed length sequences, the alphabet, the specification of the subsequence (offset, length, and sequence) to be skipped. This algorithm returns a *SBSW* with the estimated parameters.

train.txt
```
training_algorithm="SBSW"
training_set="donor.fasta"
alphabet=("A", "C", "G", "T")
skip_offset=3
skip_length=2
skip_sequence="GT"
```

5.16 Using model selection when training a probabilistic model

Many models would have different dimensionality which are defined by the user during the training procedure. Typical example includes Markov chain models in which the user has to choose the value of the order. To help find the best set of such parameters. ToPS contains two model selection criteria that the user can use with a training algorithm.

- Bayesian Information Criteria (BIC) [Sch78]: *This criteria selects the model with the largest value for the formula below:*

$$\log(Maximum\ Likelihood) - \frac{1}{2}(number\ of\ independently\ adjusted\ parameters) \times \log(sample\ size)$$

- Akaike Information Criteria (AIC) [Aka74]: *This criteria selects the model with the smallest value for the formula:*

$$(-2)\log(Maximum\ Likelihood) + 2(number\ of\ independently\ adjusted\ parameters)$$

To run a model selection procedure the user have to specify four arguments:

- *model_ selection_ criteria* specifies the model selection criteria: BIC, or AIC.
- *begin* specifies the set of parameters to be tested and their initial values.
- *end* specifies the final values for the parameters specified above
- *step* specifies the increment on the values of each of the parameters being tested.

For example, the file `bic.txt` specifies that ToPS will use BIC selection criteria. The training procedure will calculate the BIC values for the estimated VLMC for each cut in the set $\{0.0, ..., 1.0\}$, and it will return the model with the preferred BIC value.

bic.txt
```
training_algorithm = "ContextAlgorithm"
training_set = "sequences.txt"
model_selection_criteria = "BIC"
begin = ("cut": 0.0)
end = ("cut": 1.0)
step = ("cut": 0.1)
alphabet = ("0", "1")
```

Chapter 6

Simulating Probabilistic Models

ToPS provides a program, called *simulate*, which samples sequences from a probabilistic model, requires as parameters the length and the number of sequences to be generated. For example, the command line below determines the generation of 10 sequences, each with length 1000, using the HMM (hmm.txt), in standard output (screen). In this case, since we are using an HMM, the output consists of pairs of sequences (the second sequence of the pair corresponding to the hidden state labels).

Command line

```
simulate -m cpg_island.txt -n 10 -l 1000 -h
```

The command line parameters of the *simulate* program are:

- -m specifies the name of the file containing the model description.
- -n specifies the number of sequences that will be generated.
- -l specifies the length of each sequence.
- -h determines the generation of the symbols and the hidden state labels.

hmm.txt

```
model_name="HiddenMarkovModel"
state_names= ("1", "2" )
observation_symbols= ("0", "1" )
# transition probabilities
transitions = ("1" | "1": 0.9;
                         "2" | "1": 0.1;
                         "1" | "2": 0.05;
                     "2"| "2": 0.95 )
# emission probabilities
emission_probabilities = (
                       "0" | "2" : 0.95;
                       "1" | "2" : 0.05;
                       "0" | "1" : 0.95;
                       "1" | "1" : 0.05)
initial_probabilities= ("1": 0.5; "2": 0.5)
```

The simulate program is not limited to the use with HMM, any probabilistic model description works as an input.

Chapter 7

Evaluating probabilities of a sequence

ToPS provides a program, called *evaluate*, which calculates the probability of sequences given a probabilistic model, requires as parameters the model description and a set of sequences. For example, the command line below determines the probabilities of a set of sequences (sequences.txt) given the HMM model.

Command line

```
evalulate -m hmm.txt < sequences.txt
```

The command line parameter of the *evaluate* program is:

- -m specifies the name of the file containing the model description.

hmm.txt

```
model_name="HiddenMarkovModel"
state_names= ("1", "2" )
observation_symbols= ("0", "1" )
# transition probabilities
transitions = ("1" | "1": 0.9;
                           "2" | "1": 0.1;
                           "1" | "2": 0.05;
                      "2"| "2": 0.95 )
# emission probabilities
emission_probabilities = (
                         "0" | "2" : 0.95;
                         "1" | "2" : 0.05;
                         "0" | "1" : 0.95;
                         "1" | "1" : 0.05)
initial_probabilities= ("1": 0.5; "2": 0.5)
```

The evaluate program is not limited to the use with HMM, any probabilistic model description works as an input.

Chapter 8

Other Applications

Here we describe other functionalities of ToPS.

8.1 Aligning Using Pair HMM

8.2 Bayesing Classifier

When we have a set of pre-defined sequence families each specified by a different probabilistic model, we can use a Bayesian classifier to decide to which family a given sequence belongs. For each sequence, the Bayesian classifier selects the family that corresponds to the model with the highest posterior probability. In ToPS, the program *bayes_ classifier* implements the Bayesian classifier. Based on a configuration file containing a list of specified probabilistic models and the *a priori* probabilities, this program reads sequences from the standard input and returns, for each sequence, the posterior probabilities of each model. In box *bayes_ classifier.txt*, we show an example of such a configuration file. Using our CpG island example we can model it with two Markov chains [DEKM98], one to characterize CpG islands and another to characterize general genomic sequences. Then we can build a Bayesian classifier using the two models, and apply this classifier to candidate sequences. We will need then first to describe two Markov models, train each one, and with the trained files, build a classifier:

bayes_classifier.txt

```
classes =
 ( "CPG": "cpg_island_markov_chain.txt";
   "NONCPG": "uniform_markov_chain.txt")
model_probabilities =
 ( "CPG": 0.5;
   "NONCPG": 0.5)
```

The program reads from standard input and prints to standard output, so a sample would be:

Command line

```
bayes_classifier -c bayes_classifier.txt \
 < sequences.in
```

sequence name	$logP(S\|CPG)$	$P(CPG\|S)$	$logP(S\|NONCPG)$	$P(NONCPG\|S)$	classification
seq1	-141.029	0.0831703	-138.629	0.916827	NONCPG
seq2	-132.381	0.9981	-138.629	0.00192936	CPG

Table 8.1: *An example of bayesian_ classifier's output.*

The program output is a table in CSV (comma separated values) format, which is compatible with most spreadsheet programs. The rows are showing, from left to right the name of each sequence, the log-likelihood of the sequence given each model, the *a posteriori* probabilities of each model, and the predicted classification of the sequence. An example of result produced by the command above is at Table 8.1.

8.3 Sliding Window

8.4 Viterbi Decoding and Posterior Decoding

With probabilistic models for which the states do not correspond to individual symbols, decoding is an essential part of the recognition problem. In ToPS, decoding uses the Viterbi algorithm [Rab89], implemented by the program *viterbi_ decoding*. In this case, the input model is an HMM or a GHMM. With the command line below, the program *viterbi_ decoding* reads the file *in.txt* and, using the model specified in the file *cpg_ island* generates the sequence of states visited in the most probable path of the model for each sequence. The result is presented in standard output.

Command line
```
viterbi_decoding -m cpg_island.txt < in.txt
```

The command line parameter for the *viterbi_ decoding* program is:

- -m specifies the file containing the model.

ToPS also implements the posterior decoding algorithm that provides the more probable state for each position of the sequence.

Command line
```
posterior_decoding -m cpg_island.txt < in.txt
```

The command line parameter for the *posterior_ decoding* program is:

- -m specifies the file containing the model.

Chapter 9

Design and Implementation

The object-oriented architecture of ToPS was essential for the seamless integration of the models in a single system that included facilities for training, simulating, decoding, integration in GHMMs and construction of Bayesian classifiers.

ToPS's architecture includes three main class hierarchies: *ProbabilisticModel*, to represent model implementations; *ProbabilisticModelCreator*, to specify the on-the fly creation of models based on configuration files; and *ProbabilisticModelParameterValue*, to enable the parsing of the configuration files. These three hierarchies are used by a set of 13 application programs that implement the framework's user functionalities (*align, bayes_ classifier, choose_path, evaluate, kullback_positional, mea _decoding, posterior_ decoding, posterior_probabilities, pred_ align, simulate, simulate_ alignment, train, viterbi_ decoding*) .

9.1 ProbabilisticModel hierarchy

In ToPS every model is implemented as part of ProbabilisticModel hierarchy, shown in Figure 9.1. At the root of the hierarchy we have the abstract class *ProbabilisticModel*, with methods that characterize any generative model. In particular, *ProbabilisticModel* has two important abstract methods: *evaluate*, and *choose*. The *evaluate* method, in subclasses, should calculate the probability of a sequence given the model and the *choose* method should sample a new sequence given the model. This class is used by other parts of the system to ensure that new probabilistic models are included seamlessly in the framework and benefit from all current functionality.

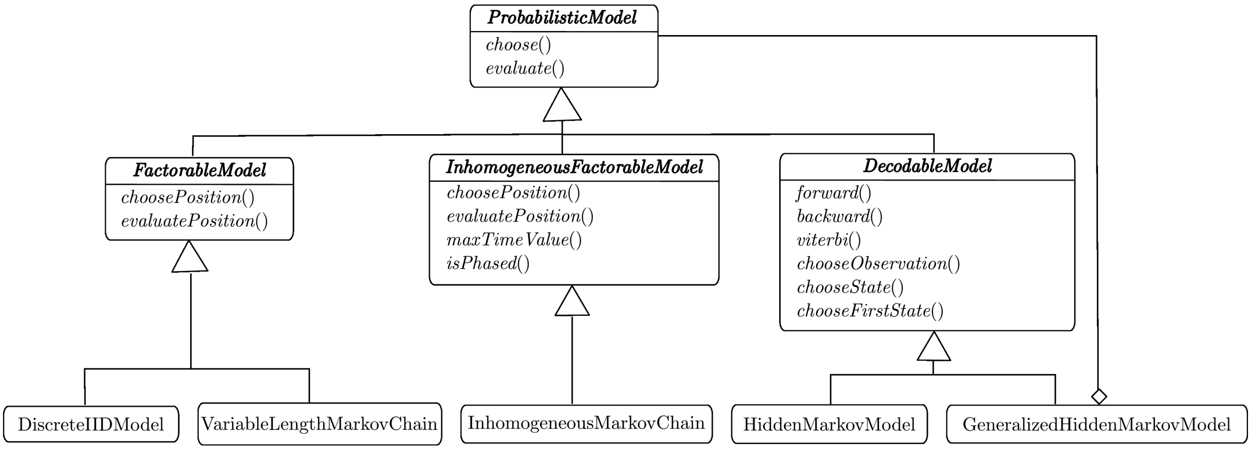

Figure 9.1: Class diagram representing the *ProbabilisticModel* hierarchy.

At the second level of the hierarchy, there are three other abstract classes that implement both the *choose* method, and the *evaluate* method: *FactorableModel*, *InhomogeneousFactorableModel*, and *DecodableModel*.

The last level of the hierarchy contains the implementation of the models currently available in ToPS in the form of concrete classes: *DiscreteIIDModel*, *VariableLengthMarkovChain*, *InhomogeneousMarkovChain*, *HiddenMarkovModel*, *PairHiddenMarkovModel* and *GeneralizedHiddenMarkovModel*. In particular, *GeneralizedHiddenMarkovModel* uses the *Composite* pattern [GHJV94], which guarantees that any new probabilistic model incorporated into the hierarchy can be used as a submodel of GHMMs. The implementation of new models will involve extending this hierarchy, and implementing the abstract methods. Using this hierarchy guarantees that new models will work smoothly with the other components of the framework.

In the next sections we will describe each of the main remaining classes of the hierarchy.

9.1.1 FactorableModel

In *FactorableModel*, the *evaluate* method calculates the following formula:

$$Prob(S|FactorableModel) = \prod_{i=1}^{L} f(S, i)$$

In which f is a function of a sequence S and a position i of that sequence. The *choose* method will generate a new sequence by sampling each symbol using the distribution given by the function f.

FactorableModel also includes two abstract methods: *choosePosition* and *evaluatePosition*. The *choosePosition* receives a sequence and a position i and samples a new symbol for the position i of the sequence using the function f. The *evaluatePosition* receives a sequence and a position i and it returns the probability of the symbol at position i of the sequence using the function f. Examples of "factorable models" are:

- *VariableLengthMarkovChain*, which can capture variable-length dependencies. The function f in this case is equal to:

  $$f_{VLMC}(s, i) = p(s_i|s_{i-1} \cdots s_{i-l})$$

  Where $p(s_i|s_{i-1} \cdots s_{i-l})$ is the probability of the symbol s_i given the past sequence, and $l = l(s_{i-1}, \cdots, s_1)$ is itself a function for the past.

- *DiscreteIIDModel*, which is equivalent to order zero Markov chains, where $l = 0$. The function f for this model is:

  $$f_{DiscreteIID}(s, i) = p(s_i)$$

  Where $0 \leq p(\sigma) \leq 1$ is the probability of the letter $\sigma \in \Sigma$.

Although *DiscreteIIDModel* is equivalent to the zero order Markov chain, we have chosen to independently implement it, because it provides more efficient algorithms for sampling symbols when $l = 0$. We have also implemented training procedure that generates other types of Markov models:

- Fixed Length Markov Chains of order k can be implemented as a *VariableLength-MarkovChain* where $l = k$ for all the past sequences.
- Interpolated Markov Model of order k is a Fixed Length Markov Models of order k, in which the probability of observing s_i for context patterns $s_{i-k} \cdots s_{i-1}$ was estimated by interpolating the estimates of smaller context patterns $s_{i-j-k} \cdots s_{i-1}$ $(0 \leq j \leq k+1)$ [SDKW98].

9.1.2 InhomogeneousFactorableModel

This abstract class represents models that are time-inhomogeneous, and factorable. In this class the *evaluate* method calculates the following formula:

$$Prob(S|InhomogeneousFactorableModel) = \prod_{i=1}^{L} g_t(S,i) \tag{9.1}$$

where g is a function of the sequence S, a position $i \in \{1, \cdots, L\}$, and a time $t \in \{0, \cdots, M-1\}$ ($M-1$ is the maximum value of t). The *choose* method will sample a new sequence by sampling each symbol for a specific time using the distribution given by the function g. Times can be used, for example, to determine different distributions for each codon position in a DNA sequence.

ToPS currently implement only one subclass of the InhomogeneousFactorableModel, *InhomogeneousMarkovChain* for which the g function is:

$$g_t(s,i) = p_t(s_i|s_{i-1} \cdots s_{i-l})$$

here $p_t(s_i|s_{i-1} \cdots s_{i-l})$ is the probability of the symbol s_i for the time t and given the past sequence. The time $t = t(i)$ is itself a function for the position, $t(i) = (i-1) \mod M$, and order $l = l(s_{i-1} \cdots s_1)$ is itself a function for the past.

Inhomogeneous Markov chains are widely used in Bioinformatics, of which the most notable examples are:

- **Weight Matrix Model** [Sta84], which is an *InhomogeneouMarkovChain* of order zero, $l = 0$ for all the past sequences.
- **Weight Array Model** [ZM93], which is an *InhomogeneousMarkovChain* with order greater than zero, $l = k$ for all the past sequences, k is a constant greater than zero.
- **Three-periodic Markov Chain** [BM93], which is an *InhomogeneousMarkovChain* with M equals to three and isPhased is true.

ToPS provides specific training algorithms to create any of these models as an *InhomogeneousMarkovChain*.

9.1.3 DecodableModel

This abstract class represents models that are used to decode sequences. Hidden Markov Model and Generalized Hidden Markov Model are examples of decodable models. There are three important algorithm for these models:

- **forward** algorithm, that calculates the probability $\alpha_k(i)$ of observing the sequence $s_1 \cdots s_i$ and ending in state k at position i.

- **backward** algorithm, that calculates the probability $\beta_k(i)$ of observing the sequence $s_{i+1} \cdots s_L$ given the state k at position i.
- **viterbi** algorithm, that calculates the probability $\gamma_k(i)$ of the most probable path ending in state k with observation $s_1 \cdots s_i$.

In this class, the methods *forward*, *backward*, *viterbi*, *chooseObservation*, *chooseState*, and *chooseFirstState* are abstract methods.

The *evaluate* method is implemented by using the result of the *forward* algorithm, returning the value:

$$Prob(S|DecodableModel) = \sum_k \alpha_k(L) f(k) \tag{9.2}$$

where $f(k)$ is the probability of terminating in state k.

ToPS implements two *DecodableModels*: Hidden Markov Model and Generalized Hidden Markov Model.

9.2 ProbabilisticModelCreator and ProbabilisticModelParameterValue hierarchies

The *ProbabilisticModelCreator* abstracts the process of instantiating members of *ProbabilisticModel* hierarchy from configuration files, implementing the *Factory Method* design pattern [GHJV94]. Training algorithms are concrete classes of this hierarchy. The *ProbabilisticModelParameterValue* was designed to facilitate the parsing of configuration files and allow them to be close to the mathematical notation of the models. This hierarchy includes 8 concrete classes that can be examined in the system's webpage.

Additionally, ToPS uses two classes *Symbol*, and *Alphabet* that enable the use of any arbitrary discrete input values by the models. Therefore the user can design system that can use as input, nucleotides, aminoacids, codon names, etc.

Bibliography

[Aka74] H Akaike. A new look at the statistical model identification. *IEEE transactions on automatic control*, AC-19:716–723, Dec 1974.

[BM93] M. Borodovsky e J. McIninch. Genmark: Parallel gene recognition for both DNA strands. *Computer Chem*, 17:123—133, 1993.

[Bur97] C. Burge. *Identification of genes in human genomic DNA*. Tese de Doutorado, Stanford University, 1997.

[DEKM98] R. Durbin, S. R. Eddy, A. Krogh e G. Mitchison. *Biological sequence analysis: Probabilistic models of proteins and nucleic acids*. Cambridge University Press, 1998.

[GHJV94] E. Gamma, R. Helm, R. Johnson e J. Vlissides. *Design Patterns: Elements of Reusable Object-Oriented Software*. Addison Wesley, Massachusetts, 1994.

[GL08] Antonio Galves e Eva Löcherbach. Stochastic chains with memory of variable length. *arXiv*, math.PR, Apr 2008. 17 pages.

[Gué03] Y. Guédon. Estimating hidden semi-Markov chains from discrete sequences. *Journal of Computational and Graphical Statistics*, 12(3):604–??, Setembro 2003.

[KD06] A. Klapuri e M. Davy. *Signal processing methods for music transcription*. Springer-Verlag New York Inc, 2006.

[KHRE96] D. Kulp., D. Haussler., M. G. Reese. e F. H. Eeckman. A generalized hidden Markov model for the recognition of human genes in DNA. *Proc Int Conf Intell Syst Mol Biol*, 4:134–142, 1996.

[MS99] C.D. Manning e H. Schütze. *Foundations of statistical natural language processing*. MIT Press, 1999.

[Rab89] L. R. Rabiner. A tutorial on Hidden Markov Models and selected applications in speech recoginition. Em *Proccedings of the IEEE*, volume 77, páginas 257–286, February 1989.

[Ris83] J. Rissanen. A universal data compression system. *Information Theory, IEEE Transactions on*, 29(5):656–664, 1983.

[Sch78] Gideon Schwarz. Estimating the dimension of a model. *The Annals of Statistics*, 6(2):461–464, Mar 1978.

[SDKW98] S. Salzberg, A. L. Delcher, S. Kasif e O. White. Microbial gene identification using Interpolated Markov Models. *Nucleic Acids Research*, 26:544–548, 1998.

[She04] S.J. Sheather. Density estimation. *Statistical Science*, 19(4):588–597, 2004.

[Sta84] R. Staden. Computer methods to locate signals in nucleic acid sequences. *Nucleic Acids Res*, 12:505–519, 1984.

[Sta03] M. Stanke. *Gene prediction with a hidden Markov model.* Tese de Doutorado, Universität Göttingen, 2003.

[SW03] M. Stanke e S. Waack. Gene prediction with a hidden Markov model and a new intron submodel. *Bioinformatics*, 19 Suppl 2:II215–II225, Oct 2003.

[ZM93] M. Q. Zhang e T. G. Marr. A weight array method for splicing signal analysis. *Computer Applied in Bioscience*, 9:499—509, 1993.
